# Genome-Wide Interrogation of Mammalian Stem Cell Fate Determinants by Nested Chromosome Deletions

**DOI:** 10.1371/journal.pgen.1001241

**Published:** 2010-12-09

**Authors:** Simon Fortier, Mélanie Bilodeau, Tara MacRae, Jean-Philippe Laverdure, Valeria Azcoitia, Simon Girard, Jalila Chagraoui, Nancy Ringuette, Josée Hébert, Jana Krosl, Nadine Mayotte, Guy Sauvageau

**Affiliations:** 1 Molecular Genetics of Stem Cells Laboratory, Institute of Research in Immunology and Cancer (IRIC), University of Montreal, Montreal, Quebec, Canada; 2 Division of Hematology and Leukemia Cell Bank of Quebec (BCLQ), Maisonneuve-Rosemont Hospital, Montreal, Quebec, Canada; The Jackson Laboratory, United States of America

## Abstract

Understanding the function of important DNA elements in mammalian stem cell genomes would be enhanced by the availability of deletion collections in which segmental haploidies are precisely characterized. Using a modified Cre-*loxP*–based system, we now report the creation and characterization of a collection of ∼1,300 independent embryonic stem cell (ESC) clones enriched for nested chromosomal deletions. Mapping experiments indicate that this collection spans over 25% of the mouse genome with good representative coverage of protein-coding genes, regulatory RNAs, and other non-coding sequences. This collection of clones was screened for *in vitro* defects in differentiation of ESC into embryoid bodies (EB). Several putative novel haploinsufficient regions, critical for EB development, were identified. Functional characterization of one of these regions, through BAC complementation, identified the ribosomal gene *Rps14* as a novel haploinsufficient determinant of embryoid body formation. This new library of chromosomal deletions in ESC (DelES: http://bioinfo.iric.ca/deles) will serve as a unique resource for elucidation of novel protein-coding and non-coding regulators of ESC activity.

## Author Summary

Stem cells have received considerable public attention in part because of their potential application in regenerative therapies. Stem cells can be operationally defined as cells that have the unique property to self-renew, as well as to generate more differentiated progeny (differentiation). However, much remains to be learned about the genes regulating stem cell differentiation and renewal, their relationship to each other, and the signaling pathways that control their expression and/or activity. In this paper, we present a new resource developed in our laboratory, called DelES, for chromosomal deletion in ES cells. By reinserting deleted DNA fragments in a set of ESC clones harboring nested chromosomal deletions, we identified the *Rps14* gene as being haploinsufficient for embryoid body formation. We think that our library of more than 1,300 clones represents a new resource that should allow the identification of genes and other elements that are essential for stem cell activity.

## Introduction

### Mammalian genomes and ESC characteristics

Mouse ESCs, derived from the inner cell mass of the blastocyst [1], [2], are a lineage of choice to perform functional genomic studies for several reasons. First, ESCs constitute a sustained source of starting material since they indefinitely self-renew symmetrically in defined culture conditions, generating two functionally identical daughter cells per division [3]. Second, pluripotent ESCs enable the study of most developmental processes *in vivo* or *in vitro*, owing to their capacity to make all somatic cell types, including germ cells [4], [5]. Third, ESCs can model various aspects of tumorigenesis. Undifferentiated ESCs are characterized by the absence of a robust G_1_/S cell cycle checkpoint [6], a feature frequently observed in tumor cells [7]. Moreover, ESCs are tumorigenic when ectopically implanted [1], [2]. Lastly, the ESCs genome is easily modifiable with various mutagenesis techniques. Because ESCs and induced pluripotent stem cells (iPS) are valuable resources for modeling human diseases *in vitro* and *in vivo* as well as a potential source for cell replacement therapy, major efforts are ongoing to decipher the molecular determinants regulating the cardinal features pertaining to these cells, such as self-renewal, pluripotency, multilineage differentiation and tumorigenic potential.

ESCs are capable of being maintained undifferentiated *in vitro* in the presence of LIF and BMP signaling [8]. Upon removal of self-renewal signals (e.g. LIF), ESCs will differentiate *in vitro* into aggregated structures called “embryoid bodies” or “EB”. ESC differentiation into EB occurs in an ordered manner, with the generation of derivatives from the 3 germ layers [9]. This feature of *in vitro* ESC differentiation seems to recapitulate, in a spatiotemporal manner, several of the differentiation processes observed *in vivo* (i.e., normal embryonic development [10]). Moreover, ESC differentiation into endoderm, mesoderm, and ectoderm is highly regulated and correlates with expression of a panel of specific markers, which can be used to characterize the extent of the differentiation process at the molecular level [11].

### Networks regulating ESC fate

Although several proteins involved in signaling, transcriptional regulation and chromatin modification are implicated in ESC activity, we still do not understand all genetic hierarchies dictating ESC fate [12]–[15]. Recent studies have also documented a function for non-coding RNAs such as microRNAs and lincRNAs in ESC behavior [16], [17].

Aside from these large classes of determinants, sequence comparison analyses suggest that other elements of the mammalian genome might be regulating biological functions, including ESC behavior. Among these elements are 480 segments of >200 bp termed “ultraconserved elements”, characterized by 100% sequence conservation (higher degree of conservation than protein-coding regions) between human, mouse, and rat genome [18]. Of these “ultraconserved” elements, more than 50% show no evidence of transcription, while others overlap with protein coding genes [18]. These sequences are enriched for homeodomain-binding modules, which is intriguing considering the important role of homeodomain transcription factors in ESC pluripotency and developmental processes [19]. Finally, although evolutionary conserved sequences may pinpoint functionally important genomic regions, other crucial elements may lack evolutionary constraints [20].

### Functional genomics in mammalian stem cells

Several large-scale functional genomics initiatives are currently ongoing to understand the molecular bases of embryonic stem cells. These include single gene inactivation (or alleles generation) using diverse strategies such as chemically-induced point mutations [21], gene/exon trapping (e.g., the international gene trap consortium: www.genetrap.org) and homologous recombination (The comprehensive knockout mouse project consortium: [22]). A repository for KOMP now exists (www.komp.org) in which 8500 genes are being targeted (several in conditional alleles) within relatively short periods of time. This repository contains several available lines from other initiatives. As a result, in mouse, most protein-coding genes will be deleted and available, many of them as conditional alleles, within the coming years. While these collections represent an outstanding resource for the community, they nonetheless leave a significant proportion of the “functional genome” unexplored. Moreover they fail to examine synthetic interaction between gene neighbours.

To complement existing resources that explore functional elements in the mammalian stem cell genomes, we have applied our recently developed retroviral tools to create a collection of ESC with nested chromosomal deletions [23]. Here we document the generation of DelES (Deletion in ES cells) library, which contains more than a thousand independent ESC clones highly enriched in chromosomal deletions and representing a large coverage of the mouse genome. Evidence is provided to demonstrate that a large proportion of these clones are competent in functional assays. A complementary method was also optimize to introduce, at high efficiency, a series of selectable marker genes in the backbone of BACs in order to rescue the inability of selected ESC clones to form embryoid bodies *in vitro*. This first validation allowed the identification of a novel gene essential for EB formation. In addition, a database was created (http://bioinfo.iric.ca/deles) to assist in sample management and to compile and interpret all genetic and phenotypic data related to the collection of clones. This database will facilitate the search for genomic regions regulating the ESC activity and the further design of rescue experiments. The library of ESC clones described herein thus has the added potential of deciphering novel determinants involved in ESC activity. On that basis, it is highly complementary to other international functional genomics initiatives.

## Results

### Generation and molecular characterization of DelES resource

**Figure 1:**
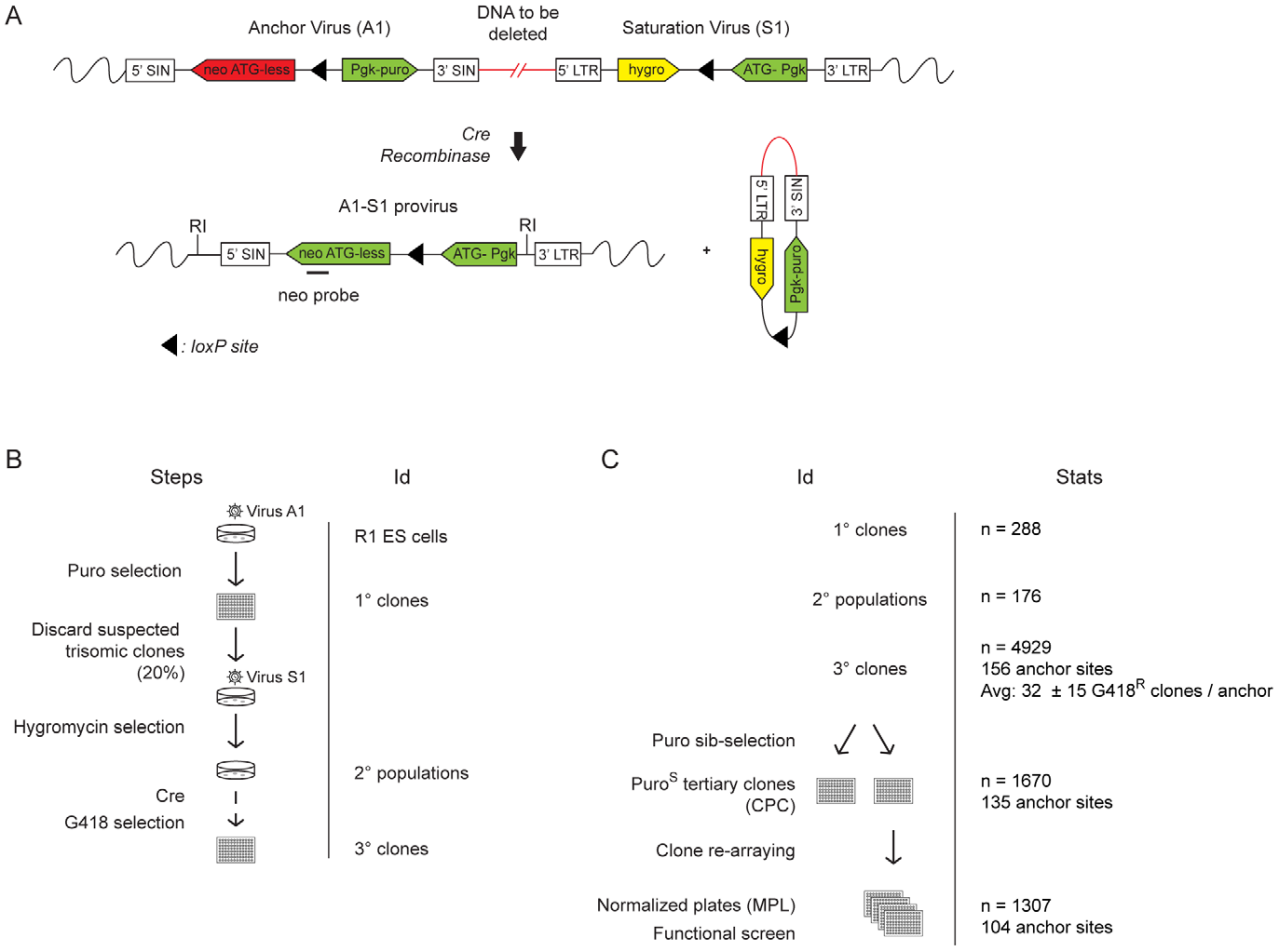
Creation of DelES resource. (A) The engineering methodology is comprised of two compatible retroviruses each containing a *loxP* sequence. The anchor virus (A1) includes a functional puromycin (puro) gene and a truncated neomycin (neo ATG-less) gene. The saturation virus (S1) includes a hygromycin (hygro) gene and a *Pgk-ATG* (murine *phosphoglycerate kinase*) promoter cassette that, following Cre recombination, drives expression of a functional neomycin gene. ESC clones harboring recombination events are selected with geneticin (G418). Chromosomal deletions lead to the concomitant loss of the puromycin and hygromycin genes. LTR, long terminal repeat; SIN, long terminal repeat containing a deletion in the U3 region. (B) Three steps were followed to generate the ESC clones in DelES: i) isolation of puromycin-resistant (puro^R^) clones containing a single A1 virus integration, referred to as primary clones; ii) infection of individual primary clones with a low titer S1 virus to generate puro^R^ and hygromycin resistant (hygro^R^) polyclonal populations of secondary clones; iii) transient Cre expression and isolation of G418-resistant (G418^R^) recombinant tertiary clones. The group of tertiary clones originating from a specific primary clone and secondary clone population is referred to as a family. (C) Summary of statistics relative to cryopreserved clones. CPC, Cherry-picked clones; MPL, master plate.

In order to generate a library of ESC clones containing nested chromosomal deletions (DelES library), we used a retroviral-based method that exploits Cre-*loxP* technology as described [23] (summarized in Figure 1A). Assisted by robotic cell culture manipulation, we upscaled the previously described procedure to generate the DelES collection (Figure 1B, see also Text S1 and Table S1). Statistics about the various groups of clones in DelES (primary, secondary and tertiary clones) and the types of chromosome rearrangements (e.g., nested chromosomal deletions) are detailed in Figure 1C. A total of 4929 G418^R^ tertiary clones (i.e., ESC clones harboring recombination events are selected with geneticin) originating from 156 anchor sites (i.e., families) were isolated (Figure 1C and Table S1). Of these, 33.8% (n = 1670) were sensitive to puromycin (puro^S^) of which 78.3% (n = 1307) were cryopreserved in 96 well plates. Previous work has shown an expected 80% chromosomal deletion rate in puro^S^ clones [23].

**Figure 2:**
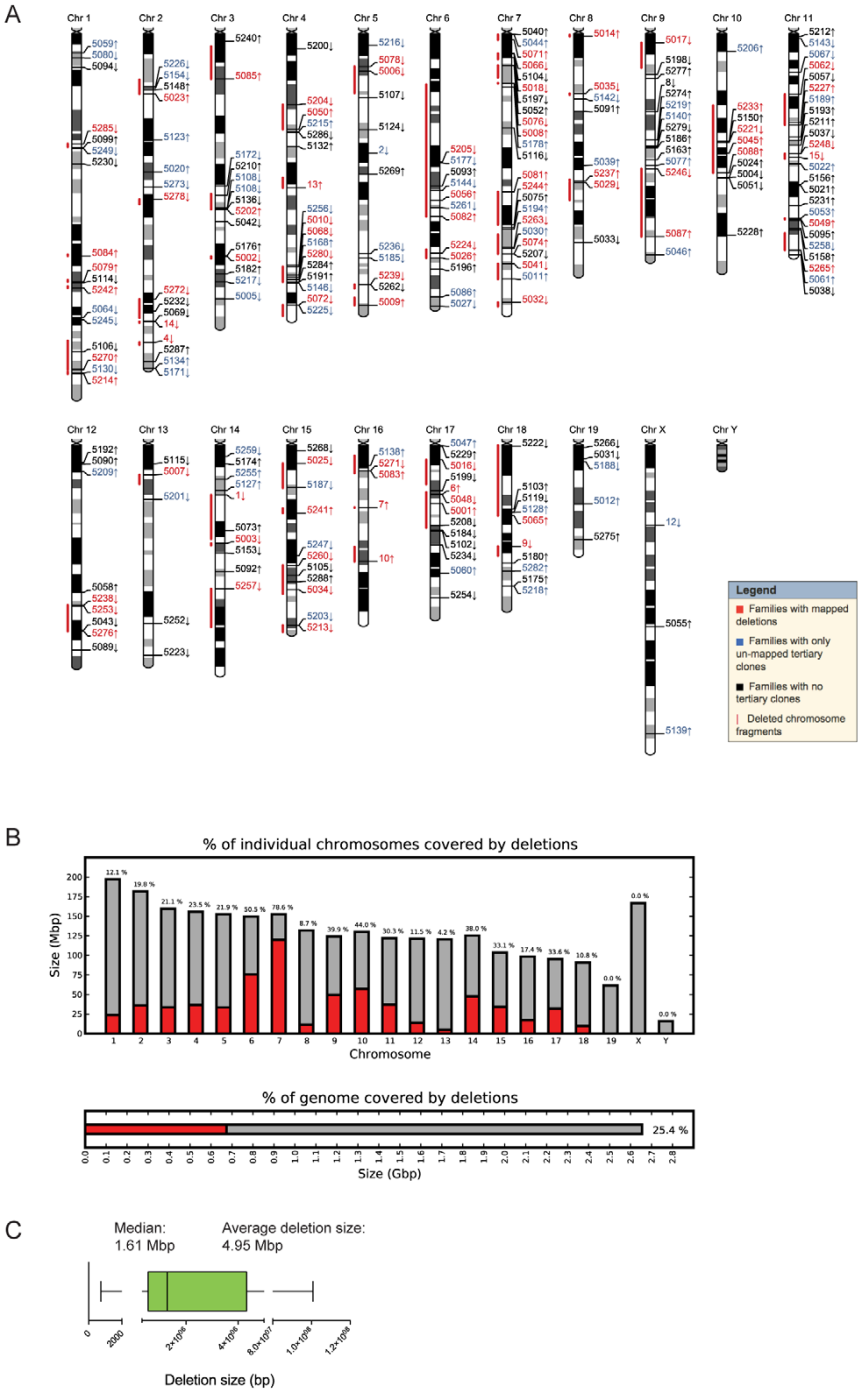
Molecular characterization of DelES clones. (A) Karyoview representation of anchor virus integration site and coverage of the longest deletion (red bar) mapped for each family of clones. The arrow indicates the predicted deletion orientation relative to the specific anchor site. (B) Genome-wide coverage of deletions mapped in DelES clones. Haploid deletions are mapped to most autosomes, covering more than 25% of the murine genome. (C) Box plot representing the size distribution associated to 95% of mapped deletions. Average and median deletion sizes are indicated.

When further characterized for proviral integration patterns by Southern blot analyses, we found that these 1307 independent clones harbored in fact 512 distinct chromosomal rearrangements (referred to as sub-families, not shown). Moreover, each family, characterized by a common anchor site, presented an average of 5.39 (range 1 to 20) distinct chromosomal rearrangements (Table S2). So far 423 deletions of which 294 are unique have been mapped by inverse or ligation mediated PCR (Figure 2A), representing 25.4% of the mouse genome (Figure 2B). The genomic coverage varies by chromosome, with no identified deletions on chromosomes 19, X or Y; limited coverage of chromosomes 8 and 13 (8.7% and 4.2%, respectively); and approximately 50% coverage of chromosomes 6 and 18 (Figure 2B). On average, there is approximately 23% genome coverage per autosome. Deletion sizes range from 736 bp to 100.79 Mb, with a median of 1.61 Mb (average size: 4.95 Mb) (Figure 2C), and vary according to the chromosome (Table S3). Chromosomes 1, 8 and 16 are characterized by many small deletions, while chromosomes 18 and 14 have a few large deletions (DelES database, http://bioinfo.iric.ca/deles).

As depicted in Figure 1A and detailed earlier [23], deletions are typically characterized by G418^R^ clones which have lost the hygromycin and the puromycin genes. Interestingly, we found 29 families in which none of the G418^R^ clones had lost hygromycin and/or puromycin resistance genes. One possibility that could explain this observation is that the anchor virus may have integrated in the vicinity of haplolethal loci. The Table S4 provides a list of genes present in the vicinity of 9 independent anchor loci that are not associated with chromosomal deletions (e.g. 9 families which had a minimum of 15 G418^R^ tertiary clones but no puro^S^ hygro^−^ clones). A literature search was performed to identify candidate genes in these regions which are known or predicted to be haploinsufficient or imprinted (in red). On average, ∼1 haploinsufficient/imprinted candidate gene was identified per 1.61 Mbp window (median deletion size) starting from each directional anchor site (Table S4). These candidate genes, alone or in combination with other genes or non-coding elements within these regions, could potentially regulate essential cellular functions or specific characteristics of ESCs and thus cannot be maintained in a heterozygous state.

Taken together, these results reveal that close to 300 independent deletions exhibiting a genome-wide distribution have been confirmed in the DelES collection.

### Genomic coverage of DelES

To evaluate the content of DelES genomic coverage, genes included in currently mapped deletions were classified according to their gene ontology (GO) terms. Gene ontology analysis of molecular functions of the 7083 mapped deleted genes revealed similar percentages in each category to that obtained for all annotated MGI genes (with known functions) (data not shown). When genes were grouped by molecular function, the most abundant group was genes with signal transduction activity, followed by transcriptional regulation and nucleic acid binding activities (data not shown).

**Figure 3:**
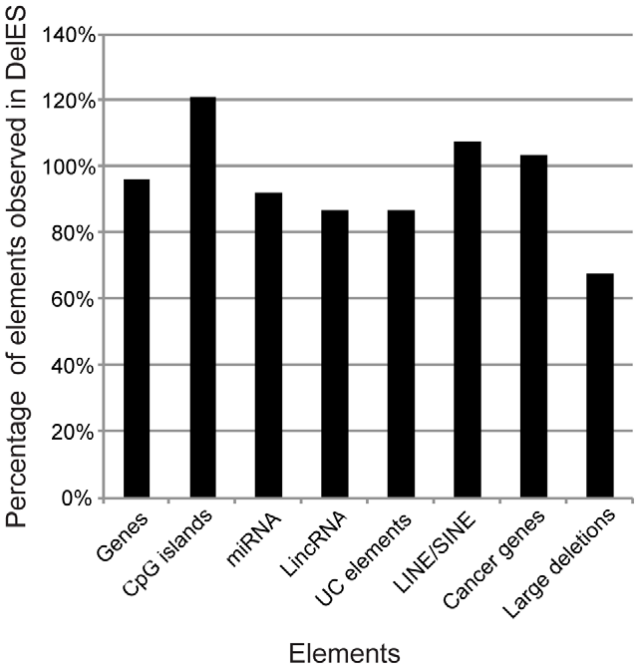
Genomic coverage of DelES. According to a hypothetical random distribution of elements throughout the murine genome, the indicated features mapped in DelES deletions were proportionally represented. Numbers used for the calculation were extracted from the following sources: MGI [49] for genes, miRBase13 [43] for miRNAs, Ensembl for CpG islands and LINE/SINE [44], the Wellcome Trust Sanger Institute website [50] for cancer related genes and large deletions linked to cancer, and a previous report [18] for ultraconserved (UC) elements.

Distribution of some key genomic elements covered by mapped deletions, such as protein-coding genes, CpG islands, miRNA, ultraconserved elements, lincRNA, LINE/SINE elements, cancer-related genes and large deletions associated with cancers was evaluated (Figure 3, Table S5). For this analysis, elements found in all of DelES's mapped deletions were compared to publicly available genome-wide entries for each category. Percentages represent ratios between the number of observed elements (found in DelES mapped deletions) and the number of reported entries (assuming random distribution of elements), based on the current genome coverage of DelES (25.4%). Interestingly, mapped deletions cover close to 100% of each category: genes (7083/7348), CpG islands (4265/3515), miRNA (128/139), lincRNA (470/540), ultraconserved elements (241/277), LINE/SINE (648571/602886), cancer related genes (108/104) and large cancer-associate deletions (5/7). Thus, several categories of protein-coding and non-coding sequences are well represented in deletions that are currently mapped. Moreover, clustering of specific genomic determinants has been reported [18], [24]–[26]. As expected, several clusters of protein coding and non-coding elements were deleted in DelES clones (highlighted in red, Table S5). Deletions of entire clusters represent another strength of DelES, as it presents the opportunity to analyze synthetic interactions between family members and to study possible functional redundancies.

### Organization of DelES collection in a public database

In order to facilitate access to the DelES collection and all clone-specific information, a database accessible through a web interface offering data mining tools was constructed (Figure S2, http://bioinfo.iric.ca/deles). A detailed explanation of the content of the interface can be found in Figure S2 and in Text S1.

Taken as a whole, the DelES database allows for the management of biological material (*Plate* tab) and facilitates the search for ESC clones through phenotypic or genetic annotations (*Selection* tab). The results of the search are directly linked to the complete data sets (*Families* tab, Figure S2). Phenotypic information can rapidly be associated to a graphical representation (*Screen* tab) of the mapped deletion including the implicated genomic features and BACs available for complementation studies (e.g., family 9).

### Quality control of DelES clones

Primary and tertiary clones in the DelES collection are distributed and frozen in 96 well plates. Localization of each clone within plates can be directly visualized online under the plate collection tab (http://bioinfo.iric.ca/deles). Colored well images indicate the presence of suspected undesired chromosomal anomalies and rearrangements (e.g., detection of hygromycin gene). Master plates containing DelES collection were thawed once to rule out microbial contamination and to determine the proportion of clones which proliferate normally (e.g. high proportion of Ki67^+^ cells) or those which express high levels of alkaline phosphatase activity, typically associated with undifferentiated ESC.

**Figure 4:**
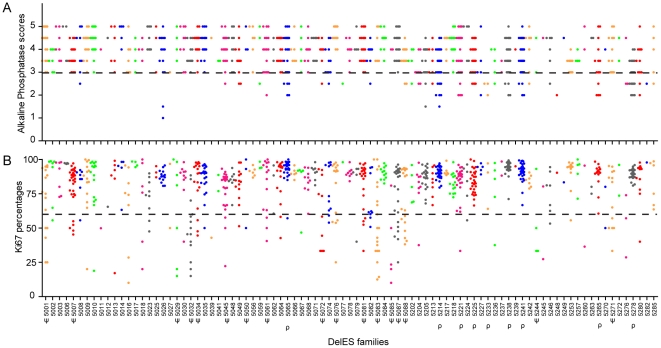
Quality control of DelES clones. 1080 puro^S^ hygro^−^ tertiary clones, grouped in 89 independent families, were analyzed for alkaline phosphatase (AP) staining (self-renewal/pluripotency) and expression of Ki67 (proliferation). (A) AP scores, ranging from 1 to 5 (1 = weak staining; 5 = strong staining), represent the mean values of one experiment performed with two independent cell densities. Approximately 9% of clones (9 families with more than two clones) demonstrated an abnormal phenotype for this assay (dashed bar represents the abnormal phenotype cutoff, arbitrarily set at 3). (B) Ki67 values are expressed as the percentage of Ki67-positive cells, gated on viable cell populations by flow cytometry. Approximately 14% of the clones (17 families with more than 2 clones) presented a proliferation defect (dashed bar represents the abnormal proliferation cutoff, stringently set at 60% Ki67-expressing cells). Families with at least 3 clones harboring an abnormal phenotype are marked by the following symbols: ρ or ψ, for AP or Ki67 category, respectively.

Figure 4A shows that nine percent of the puro^S^ hygro^−^ tertiary clones showed low alkaline phosphatase activity (scores <3; families with clusters of clones with low AP activity are identified as ρ in Figure 4), suggesting that their pluripotent capabilities were impaired. Specific genomic features covered by the nested deletions in these cells may be responsible for maintaining the pluripotent state of ESCs. This possibility will be investigated separately as part of a screen which will include multilineage differentiation assays and additional markers of pluripotency, such as Oct4.

Figure 4B shows that 14% of puro^S^ hygro^−^ tertiary clones presented low levels (<60%) of Ki67^+^ cells. Clusters of clones presenting altered proliferation status (Ki67^+^ <60%) were observed in 17 families (identified as ψ, Figure 4). The quantification of Ki67 expression was highly correlated with observed cell proliferation rate measured by flow cytometry using calibrated beads (data not shown) and batch collection of clones based on cell density or expansion (i.e. clones collected in first batch “A” expand faster than those in last batch “D” which were the slowest to expand; see Figure S1 and Text S1). This observation was also validated by a cell density assay which estimated ESC colony number one day after plating (data not shown). Unfortunalely, clones with very low proportion of Ki67^+^ cells are easily lost upon freeze thaw procedures and are difficult to maintain in the collection (S.F., personnal observation).

Overall, the vast majority of clones in the DelES collection express high levels of alkaline phosphatase; they proliferate normally; they support freeze-thaw procedures and they appear free of microbial contaminant. Clones can be recovered individually (all frozen in 96 well plates and individually) from several freeze thaw cycles. This suggests that DelES clones might be amenable to different functional screening procedures and that they can be validated separately. However, clones with low Ki67 activity are difficult to maintain.

### Functional screen of DelES: *in vitro* EB formation

**Figure 5:**
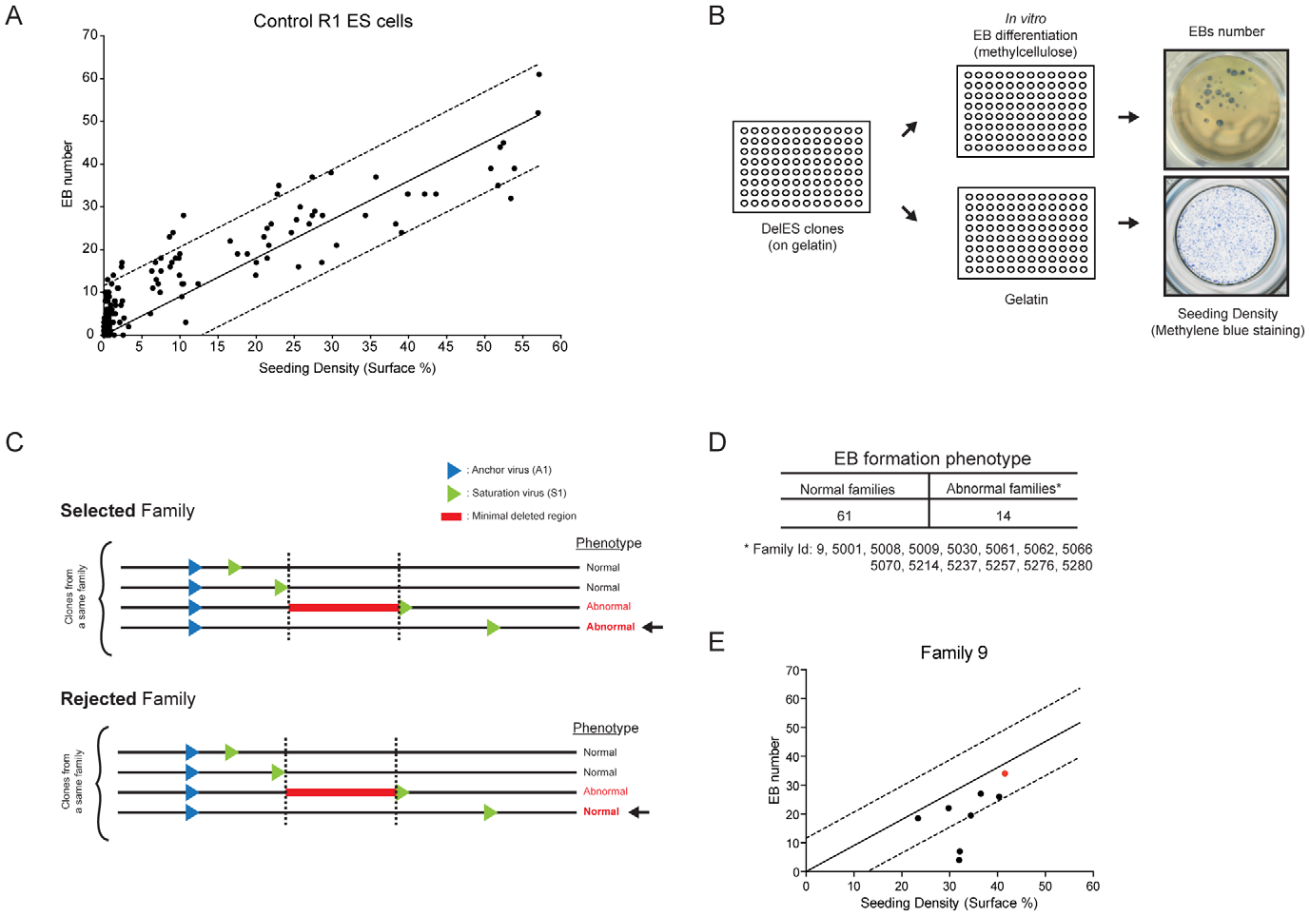
Embryoid body formation screen. (A) Correlation of R1 control ESC seeding density versus embryoid body formation Linear regression curve from control cells is shown ±1 SD (B) Screen setup. Normalized master plates were thawed on irradiated feeder cells (MEF), before being seeded on gelatinized plates prior to differentiation. After trypsinization, cells were plated in parallel, at equal volumes, in a gelatinized plate and in a 96 well plate containing semi-solid differentiation media. Cells in gelatinized plates were stained 24 hours after seeding with methylene blue (seeding density) and EB number was assessed 8 days after seeding. (C) Phenotype identification of selected vs rejected families using criteria described in text. (D) Phenotype identification in DelES families. (E) Distribution of family 9 clones in the screen setup described in B. Linear regression curve from control cells is shown ±1 SD. Outliers correspond to abnormal clones 9–18 and 9–37 which are used in complementation studies (Figure 6 and Figure 7). Red dot corresponds to primary clone 9.

Using control (parental) R1 ESCs, we observed a strong correlation between the number of EB generated in culture and the number of ESCs plated (Figure 5A). This value was reliable when cell density was above 5% (S.F., Figure 5A and data not shown). This observation was exploited to develop a functional screen in which each clone from DelES was individually seeded in two 96 well plates, one with LIF for estimation of ESC colony numbers (seeding density in Figure 5A) and the other without LIF for EB differentiation (Figure 5B), aiming to identify minimal deleted regions that cause a block in normal EB development. Three criteria were used to identify clones and families with EB formation anomalies: 1) clones were only considered if cell density was above 5% (45% of clones); 2) EB formation was considered abnormal in a tertiary clone if EB number was below one fifth of that detected in the corresponding primary clone (16.4% of clones); 3) families with EB phenotype were selected only if all clones with deletions exceeding that of the minimal deleted region also show the phenotype (see Figure 5C for selected vs rejected families). The high percentage of clones that were eliminated based on the first criteria reflects the wide distribution of cells (e.g., less than 1% to over 10%) recovered after freeze-thaw process (see also Methods for methylene blue staining). Using criteria described above, 15.6% of the families (n = 14) were considered as potentially interesting for the future identification of EB formation determinants (Figure 5D and http://bioinfo.iric.ca/deles for details of each clones in the selected families. See also Table S6 for primary screen data).

Several tertiary clones from five randomly selected families (9*, 5061*, 5035, 5214, 5238; * = families with phenotype) were tested for *in vitro* EB formation using standard assays in 60 mm dish in duplicate experiments. Corresponding primary clones were included in these validation experiments. These studies showed a concordance rate of 78% (28 out of 36 tested) between tertiary clones tested in validation experiments and the results obtained in the primary screen. In total, 3 of the 5 tested families were validated including two (9, 5061) which show putative phenotype associated with a minimal deletion region, located on chromosome 11 and 18 for family 5061 and 9, respectively (see http://bioinfo.iric.ca/deles) From this assay, it thus appears that the primary screen underestimates the frequency of families which include clones with EB differentiation phenotype. Consistent with this, our 15.6% hit rate is below that previously observed in our pilot studies of nine families (33% of families showed EB differentiation phenotype [23].

### Complementation studies of DelES family 9

**Figure 6:**
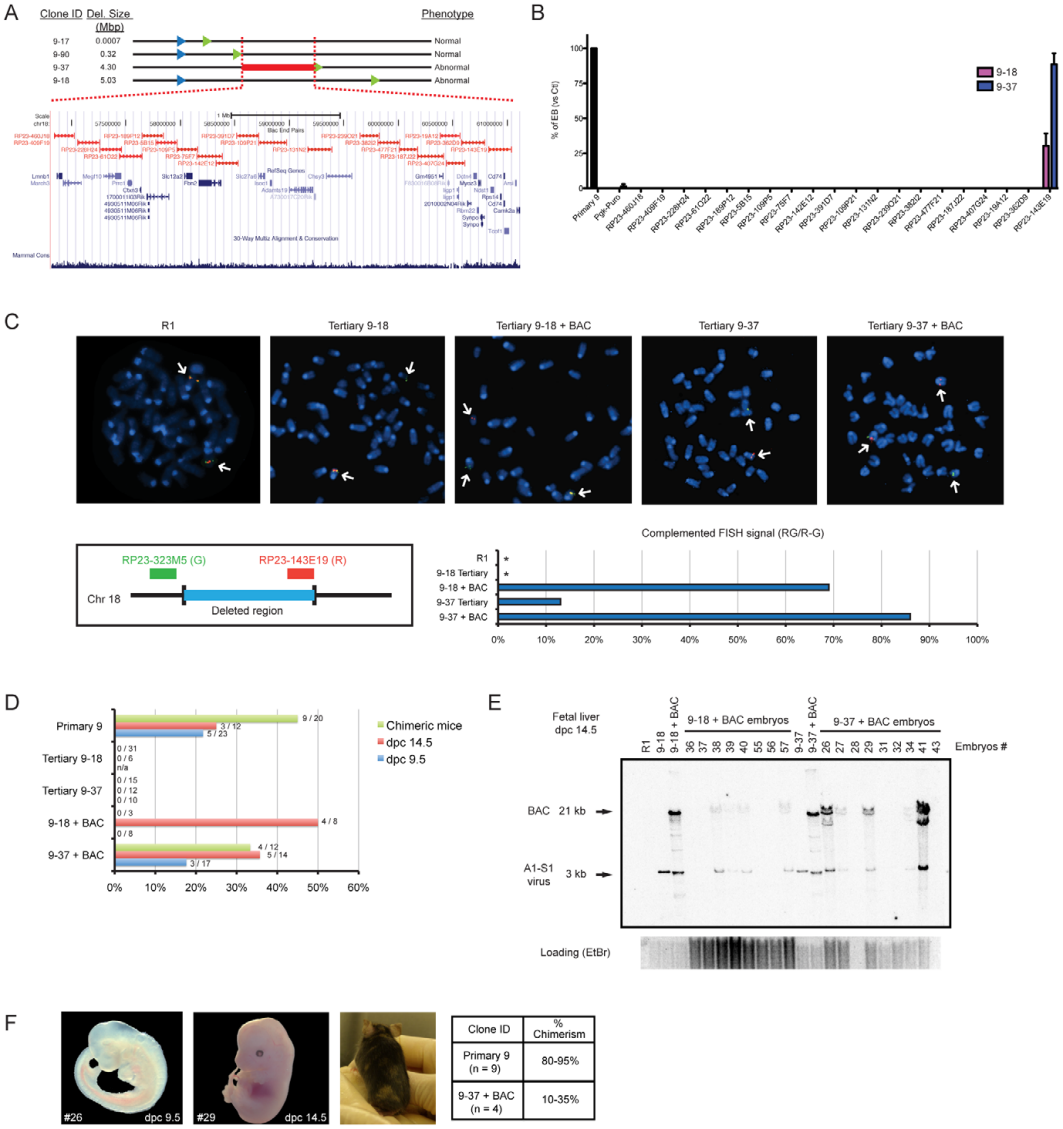
Rescue of differentiation defects of tertiary clones from DelES family 9. (A) Genomic representation of minimal deleted region leading to a differentiation phenotype in family 9 tertiary clones. Schema adapted from UCSC Genome Browser (B) Embryoid body differentiation assay following stable transfections of modified BACs covering the region of interest. Only BAC RP23-143E19 transfection could rescue the differentiation defect in tertiary clones 9–37 (complete) and 9–18 (partial). Bars represent average EB number (transfected tertiary clones normalized to control primary clone) from four independent transfections (n = 4 tests per experiment). Complete and partial complementation leads to at least 300X and 100X EB over background EB number, respectively. (C) FISH analysis of BAC-transfected clones. *Upper panel*: representative metaphase FISH images for selected clones. *Lower left panel*: FISH assay design, using two BACs as probes. RP23-323M5 is the control probe, which hybridizes outside of the deleted region, and is labeled with a green fluorescent marker (G). The BAC of interest, RP23-143E19 is labeled with a red fluorescent marker (R). *Lower right panel*: Complemented FISH signal reported as the proportion of cells which display a yellow (Red + Green: RG) signal combined with Red and Green (R–G) signals over the total number of cells analyzed (n = 100 interphase cells and n = 10 metaphase cells). *, no RG+(R–G) cell observed. (D) *In vivo* contribution of BAC-transfected and control clones to chimeric animals was evaluated by PCR for E9.5 and E14.5 embryos and by coat color chimerism for adult mice. Values are represented as proportion of neomycin positive embryos over total number of embryos analyzed and the proportion of adult chimeric mice over total number of newborns. Data related to non-transfected tertiary clone 9–18 has been previously reported [23]. (E) Southern blot analysis of DNA extracted from chimeric dpc14.5 fetal livers. *Eco*RV restriction digest combined with a *neo* probe revealed a 3 Kb and a 21 Kb fragment, corresponding to the primary anchor virus and modified BAC (*Eco*RV restriction sites in both insert and backbone), respectively. (F) Pictures of representative embryos, chimeric mouse and table of average chimerism estimated by coat color determination (newborn and adult mice). Dpc, days post coitum.

DelES family 9 was chosen as the prototype for complementation studies since clones of this family harboring large deletions are unable to differentiate into embryoid bodies. Of importance for the complementation studies described below, the frequency of EB formation in clones containing large deletions was lower than 1 in 5000 (i.e. 1 EB for 5000 cell plated). The minimal region responsible for the abnormal phenotype (e.g., red line in Figure 6A) was mapped between the breakpoints of tertiary clones 9–35 (736 bp deletion, normal *in vitro* differentiation) and 9–37 (4.3 Mbp deletion) (Figure 6A). This minimal deleted region contains 30 known protein-coding genes and can be covered by 20 contiguous bacterial artificial chromosomes BACs (Figure 6A). These 20 BACs were modified for selection in ESC using a strategy that we adapted from existing recombineering systems to allow for the introduction of selectable marker gene into the chloramphenicol resistance sequence present in the backbone of the different BACs [27]. Details about this method, called SelectaBAC, are provided in Text S1 and in Figure S3.

Two independent tertiary clones with EB formation phenotype, 9–37 and 9–18, were transfected with each of the 20 BAC constructs separately and assayed for embryoid body formation. Interestingly, none of the BAC but one -RP23-143E19- led to a complete rescue of the differentiation defect of clone 9–37, and partial rescue of clone 9–18 which contains a larger deletion (Figure 6B).

To validate the presence of transfected BAC in complemented ESCs, metaphase fluorescence in-situ hybridization (FISH) was conducted using two differentially labeled probes: the first being RP23-143E19 itself and the second a control BAC which maps adjacent to the deleted region (Figure 6C, *lower-left panel*). Transfected clones were compared with untransfected tertiary controls and R1 ESCs. As expected, normal R1 ESCs had two pairs of closely localized signals, corresponding to the intact mitotic chromosomes 18 (Figure 6C). Haploid deletions were confirmed in tertiary clones 9–37 and 9–18, which had only one pair of RP23-143E19 signals, closely-localized to one of the two pairs of control BAC signals. When clones 9–37 and 9–18 were transfected with the BAC of interest, 86% and 69% of cells counted displayed a pattern consistent with stable integration of the BAC (Figure 6C, *upper and lower-right panels*). This pattern corresponds to two pairs of RP23-143E19 signals, one on chromosome 18 identified by the control BAC signals and another on a different chromosome (not identified by the control BAC signals). Twelve percent of transfected 9–37 clones had larger and more intense red signals (RP23-143E19), which potentially indicates multiple integrations within the same chromosomal region (data not shown).

Control primary 9 clone, tertiary clones 9–18 and 9–37 and BAC-complemented tertiary clones 9–18 and 9–37 were injected separately into blastocysts or aggregated with CD1 morulas to evaluate their contribution to developing embryo. Mouse embryos, at E9.5 and E14.5 were analyzed for the presence of the neomycin gene (A1 provirus or A1-S1 recombined proviruses) by PCR, whereas the level of chimerism in newborns was estimated by coat color variation (Figure 6D). As previously reported for the clone 9–18 [23], the unmodified tertiary ESC clone 9–37 also failed to contribute to tissue chimerism in early embryos or newborn mice (Figure 6D). In contrast, primary clone 9, used as a positive control, contributed to tissue chimerism of 17 out of 55 mice analyzed (Figure 6D). RP23-143E19-transfected clone 9–37 also contributed to embryogenesis with tissue chimerism in 36% and 18% of E14.5 and E9.5 embryos, respectively, and in 4 of 12 newborn mice (Figure 6D). RP23-143E19 transfected clone 9–18 also produced chimeric embryos with a frequency of 50% at E14.5. Thus far, all chimeras (embryos and newborn) appear phenotypically normal.

Confirmation that BAC-transfected ESCs contributed to the chimeric embryos was obtained using Southern blot analyses performed with gDNA extracted from fetal liver cells (Figure 6E). For newborn and adult mice, percentage of tissue chimerism was estimated at 80–95% and 10–35% for derivatives of primary clone 9 and BAC-complemented clone 9–37, respectively (Figure 6F).

### Candidate gene evaluation for DelES family 9

**Figure 7:**
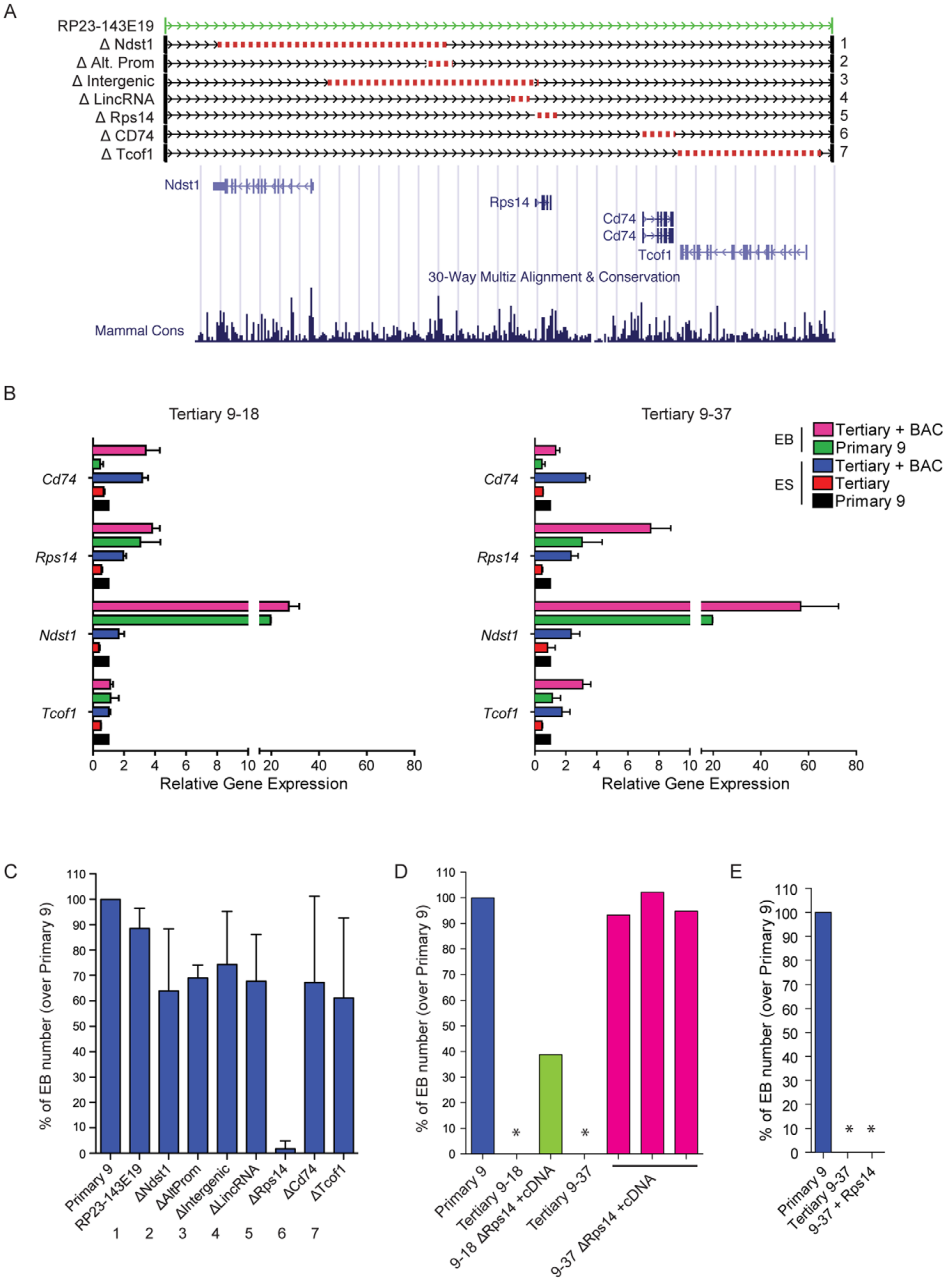
Candidate gene evaluation of DelES family 9. (A) Genomic representation corresponding to the coverage of BAC RP23-143E19 and 7 deletion constructs. Schema adapted from UCSC Genome Browser (B) Expression analysis of candidate genes included in BAC RP23-143E19. Q-RT-PCR results are represented as relative expression to that observed in family 9 primary clone (n = 4 independent experiments in triplicates). Average ΔCt per gene, for family 9 primary clone normalized with GAPDH expression levels, in ESCs and EBs, respectively; *Ndst1*: 10.85 and 6.55; *Tcof1*: 5.82 and 5.84; *Rps14*: 0.51 and 0.95; *Cd74*: 13.09 and 14.39. (C) Relative EB number derived from clone 9–37 cells previously transfected with BAC RP23-143E19 or each of the 7 deletion constructs presented in “A”. Results are compared to those obtained with identical experiments performed with the corresponding primary clone 9 (n = 4 independent experiments, in duplicate; values for primary clone 9 adjusted to 100%). (D) EB number following co-transfection of *Rps14* deletion-containing BAC plus *Rps14* cDNA. Values are represented as EB number of 3 transfected 9–37 clones and 1 transfected 9–18 clone over primary 9 clone levels. No EB was observed in cultures initiated with cells from tertiary 9–18 and 9–37 clones (*). (E) EB number following transfection of *Rps14* cDNA in tertiary clone 9–37. No EB was observed in this condition (*, n = 11 clones analyzed).

A more detailed analysis of BAC RP23-143E19 reveals the presence of four protein-coding genes: *Ndst11*, *Tcof1*, *Rps14* and *Cd74* (Figure 7A). Q-RT-PCR analyses were performed to assess the expression level of these genes in family 9 ESCs and EBs, with or without BAC RP23-143E19 complementation. All four genes analyzed are expressed in both control ESCs and EBs (primary 9) with delta CT values ranging between 0.5 (*Rps14*, highest expression) to 14.4 (*Cd74*, lowest expression) (data not shown). Expression levels of all 4 genes in tertiary clone ESCs were about half that observed in primary clone 9 (compare red bars, tertiary clones to black bars, primary clone in Figure 7B). Upon BAC transfection, expression levels of all four genes became either comparable to -or exceeded- that found in the primary clone (compare blue with black bars for undifferentiated ESCs and pink with green bars for EBs in Figure 7B).

To gain insight on the contribution of selected elements present on BAC RP23-143E19 to the observed phenotype, seven distinct deletions were generated (Figure 7A). These included deletion of all 4 protein-coding genes separately, the intergenic region between *Ndst1* and *Rps14*, a distal promoter to *Ndst1* and a lincRNA close to *Rps14*. Six out of the 7 constructs complemented the EB formation defect observed in clone 9–37 to levels comparable to control primary 9 clone. These 6 constructs complemented clone 9–18 to levels equivalent to those detected with the unmodified BAC (data not shown). Interestingly, the BAC containing the small deletion (3.89 Kb) corresponding to the *Rps14* gene did not rescue the phenotype observed in clone 9–37 (Figure 7C) and clone 9–18 (data not shown), indicating that this genomic region is haploinsufficient for EB formation.

Following this observation, *Rps14* cDNA expression vector was introduced in 9–37 and 9–18 ESC clones co-transfected with the BAC RP23-143E19 construct no. 5 (lacking the *Rps14* gene, Figure 7A), to verify the possibility that a hidden genetic element located within this small region that includes *Rps14* was responsible for the EB formation phenotype. Results from these experiments indicated that 3 out of 8 clones isolated from 9–37 doubly transfected cells showed full complementation and 1 out of 4 clones from 9–18 cells was partially rescued (Figure 7D). Expression analyses of these complemented clones revealed that all 4 protein-coding genes (*Ndst1*, *Tcof1*, *Cd74* and *Rps14*) were expressed at endogenous levels when compared to the primary clone (data not shown). These results thus strongly suggest that *Rps14* is haploinsufficient for EB formation.

We then transfected *Rps14* cDNA alone in 9–18 (data not shown) and 9–37 tertiary clones to test if this gene is the sole element responsible for the abnormal phenotype. Interestingly, analyses of several transfected clones showed no complementation with *Rps14* cDNA (Figure 7E), raising the possibility that another genetic element is necessary for complementation of DelES family 9. Nevertheless, these experiments show that *Rps14* is not sufficient, but required, to complement the EB formation defect found in DelES family 9.

## Discussion

The DelES library described herein contains a large collection of independent ESC clones harboring chromosomal deletions generated by a retroviral-based Cre-*loxP* system. Clonal analysis revealed the presence of several independent deletions in this library of which a significant proportion (close to 300) has been mapped to various locations on mouse autosomes. With a median deletion size of 1.61 megabase pairs, deletions cover more than 25% of the haploid murine genome. Overall, they include 7083 genes, of which one is reported to be haploinsufficient (*Pml*), seven are predicted to be imprinted and 108 are associated with cancer. In addition, 128 miRNAs, 241 ultraconserved elements, 470 lincRNAs and 648571 SINE/LINE elements are covered by the deleted regions (Figure 3, Table S5). The first screening test for this library consisted in analyzing EB formation upon LIF deprivation. Fourteen families exhibited an abnormal phenotype. Pertinent information of each clone in the collection is included in a public database which was created to centralize information of this expanding resource. This paper also includes the description of an optimized BAC engineering strategy which greatly facilitates complementation studies. This approach was thoroughly validated by the successful rescue of the *in vitro* differentiation defect of one family of clones, identifying *Rps14* as a novel haploinsufficient gene in EB formation from undifferentiated ESC.

To date, few genes, when disrupted, have generated overt *in vitro* differentiation defects in ESCs. For example, ESCs that are homozygous null for *smad4* will generate much fewer cystic embryoid bodies *in vitro* [28]. B-catenin activity also appears to play a critical role in ESC differentiation since *Apc* homozygous null ESCs fail to form neuroectodermal derivatives, although they form (visceral and parietal) endoderm [29]. EB formation from *ext^−/−^* (a tumor suppressor gene coding for an enzyme required for the biosynthesis of heparin sulfate) ESCs is also defective with a total absence of cavity formation, similar to the phenotype observed with *smad4* mutant cells [30]. Surprisingly, all of these genes are known for their tumor-suppressive potential in various human cancers: *smad4*, *apc* and *ext1* in pancreatic, colon and bone cancers, respectively. Although any generalization from these findings would be premature, it is tempting to speculate that additional tumor suppressor genes will be uncovered from functional screens for genes that interfere with EB formation.

Recent studies would indicate that the ribosomal protein (RP) coding gene *Rps14* identified in this study may also perform tumor suppressor functions in a preleukemia syndrome in humans [31], [32]. Indeed, this gene has been implicated in the human 5q- syndrome (now called myelodysplastic syndrome with isolated del(5q)), characterized by anemia and other cytopenias, as well as other ribosomal protein coding genes that have been linked to several blood differentiation disorders in human [32]. A p53-dependent mechanism was identified lately has being responsible of a mouse model of the 5q- syndrome. This model includes a deletion of the *Rps14* gene [33].

Ribosomes are made of large (60S) and small (40S) subunits each preassembled in the nucleolus. The large subunit is made of 3 RNA species (5S, 5.8S and 28S) and about 49 different RPs. The small subunit is made of a single 18S RNA molecule and approximately 33 RPs [34]. Recent studies suggest that a common mechanism involving defective ribosome biogenesis (also called “nucleolar stress”) may underlie the cell differentiation defect found in several human blood cell differentiation disorders, now collectively called “ribosomopathies”[35]. It was recently shown that a RPS6 partial deficiency triggers a p53 response through RPL11 interaction with MDM2, providing insights on molecular effectors of the “ribosomal stress” response [36]. Based on our findings with *Rps14*, future experiments will aim to better characterize this RP-MDM2-P53 axis in ES cells differentiation and to identify other genetic elements that contribute to our EB formation phenotype.

The assessment of haploinsufficient synthetic interactions between several contiguous genomic determinants, transcribed or not, is a feature that distinguishes chromosomal deletion engineering from other genome-wide reverse genetic approaches. Distinctively based on a precisely localizable pair of retroviral vectors, DelES is a resource complementary to other large scale methodologies that allow the creation of nested chromosomal deletions, such as: MICER [37] which is based on *loxP*-containing insertional targeting vectors, or DelBank [38] which is based on irradiation-induced deletions of integrated cassettes.

DelES clones offer many possibilities for reverse genetics since they can be used to conduct *in vitro* differentiation assays, to generate mutant animal models (chimeric, heterozygous, and homozygous animals) [39] and potentially to create teratoma/teratocarcinoma following heterotopic grafts into syngenic mice. Moreover, engineered ESCs could be used to study dominant and recessive (*in vivo*) chromosomal deletions associated with human conditions with complex phenotypic profiles that cannot be modeled using gene-specific mutagenesis approaches, such as certain cancers (4 syntenic regions covered by DelES clones). For this purpose, the correlation between chromosomal deletion size and germ line transmission rate will need further investigation.

### Conclusion

In conclusion, DelES is a new resource that offers a library of ESC deletion clones, a BAC complementation system (SelectaBAC) and a comprehensive database. DelES benefits from precisely localizable *loxP*-containing retroviral vectors which accelerate the generation of segmental haploidy, and is complementary to other functional genomics resources. Its usefulness for uncovering ESC fate determinants was demonstrated herein with the identification of *Rps14* as a novel haploinsufficient gene for EB formation and early embryonic development. DelES is designed to assess the roles of adjacent coding and non-coding sequences in the mammalian genome, as well as their genetic interactions. Our current efforts are to extend the coverage of mapped deletions in DelES clones and to conduct additional functional screens, such as cell cycle analysis, pluripotency assessment and hematopoietic differentiation, to enrich our publicly available resource.

## Methods

### Generation of DelES collection

Viral producer cell lines and infection of target cells were conducted as described [23]. Reagents used for Cre-*loxP* recombination (A1 and S1 retroviruses, and pCX-Cre constructs) were described previously [23]. Briefly, following R1 ESCs [40] infection with anchor virus A1, approximately 288 puromycin resistant primary clones were isolated. Q-PCR assays were performed on genomic DNA to discard primary clones containing presumptive trisomies. Five million primary clone cells (one clone at a time) were infected with the saturation virus S1. Following hygromycin selection, 10^7^ cells from these secondary populations (secondary population are derived from a single primary clone) were electroporated with 25 ug of supercoiled pCX-*Cre* and selected with G418, as described previously [23]. Up to 44 G418^R^ tertiary clones were isolated per electroporated secondary population and maintained in 96-well plates (labeled TER0xxx). ESCs maintained in 96-well plates were either dissociated manually or with a Biomek FX robot (Beckman Coulter) enclosed in a biosafety cabinet. G418 resistant tertiary clones sensitive to puromycin (puro^S^) were arrayed together in 96-well plates (labeled CPC0xxx). “Normalized” 96-well plates were also generated with puro^S^ clones presenting similar proliferation rate for use in functional assays. ESCs were cryopreserved at each stage of DelES collection generation. A detailed description of the methods used for the generation of DelES collection can be found in Text S1.

### High-throughput functional assays

Detailed descriptions of the high-throughput assays performed with puro^S^ clones arrayed in normalized plates are provided in Text S1. The Alkaline Phosphatase Kit (Chemicon) was used according to the manufacturer's protocol. ESCs immunostained with a PE-conjugated mouse anti-human Ki67 monoclonal antibody (dilution 1∶100, BD Biosciences) were analyzed by flow cytometry. Cell counts were performed by flow cytometry using TruCOUNT reference beads (BD Biosciences). Cell densities were evaluated by methylene blue staining of ESC colonies.

### Embryoid body formation screening

Gelatin-plated clones were seeded in 96 well plates (Sarstedt) containing a semi-solid differentiation media and in parallel on a new gelatinized plate (NUNC). EBs were counted following 8 days of differentiation, while colonies on gelatinized plates were stained with methylene blue twenty-four hours after seeding. Automated quantification of the methylene blue stained area was used to evaluate the cell input that produced the corresponding EB number (Metamorph software). Criteria were established to determine families with clones presenting abnormal EB formation phenotypes, i.e. insufficient EB number or disaggregation. The first was to exclude tertiary clones with low cell input values (methylene blue <5%, n = 722 clones, 55.2%), which could be the result of a defect in proliferation or cell adhesion, or simply a technical issue. The number of EBs obtained for each tertiary clone was compared to that of the corresponding primary clone. Based on values obtained from larger format experiments, a tertiary clone was called abnormal when it formed less than 20% EBs relative to its primary clone (n = 96 out of 585). Our final criterion in identifying an abnormal family was to verify a correlation between decreased EB formation with a larger deletion size (where mapping was available).

### DNA analyses

Genomic DNA (gDNA) from primary clones was extracted with DNeasy 96 Blood & Tissue Kit (Qiagen protocol) and used for Q-PCR screening of presumptive trisomies and mapping of proviral integration sites (see below and Text S1). Genomic DNA from primary clones, all tertiary clones (labeled TER0xxx), and puro^S^ tertiary clones (labeled CPC0xxx or MPL0xxx) were extracted using DNAzol (Invitrogen) by centrifugation in V-bottom 96-well plates. Southern blot analyses were performed as previously described [23]. To verify single integration of anchor virus, primary clone gDNA was digested with *Bgl*II-*Bam*HI restriction enzymes and detection performed with a neomycin probe. Southern blot analyses with tertiary clone gDNA (*Eco*RI restriction digest), were either performed with a neomycin probe to asses clonal diversity of rearrangements (e.g. clone classification into sub-family) or with a hygromycin probe to confirm the loss of hygromycin resistance gene. The presence/absence of hygromycin gene was also monitored by Q-PCR assays (Text S1).

### Mapping of proviral integration sites

Integration sites of the anchor virus were mapped in primary clones by I-PCR or LM-PCR. Saturation virus integration sites were mapped in tertiary clones by LM-PCR. The I-PCR approach was previously described [23]. The LM-PCR strategy, which relies on specific oligonucleotides described in Table S7, was adapted from a published protocol [41] summarized in Text S1. DNA sequences corresponding to proviral integration sites were mapped using the BLAT alignment tool of the UCSC Genome Browser (http://genome.ucsc.edu/, NCBI mouse Build 37) [42].

### Construction of database

#### Biological material tracking and database construction

Frozen cells, DNA, RNA, and maintenance plates were identified with bar codes and specific labeling (details provided in Text S1). A PostgreSQL database was set up in order to maintain a centralized repository of the biological sample's storage locations, as well as to accumulate various types of results and annotations. A web front-end running on a Webware for Python application server was also developed to enable a user-friendly access to the majority of the data contained in the database. Numerous visualization, data-mining, and sample management tools are still under development to provide a flexible interface to query the annotations and to manage access to the biological samples. Genome annotations presented in some of DelES' visualization tools as well as the gene searching capabilities rely on locally deployed instances of mirBASE [43] (http://microrna.sanger.ac.uk/) and Ensembl [44] (http://www.ensembl.org/index.html). Published LincRNA [16] and Ultraconserved Elements [18] annotations were also inserted into the DelES database to facilitate integration. More functionalities of DelES database are described in Text S1.

### BAC engineering

BACs from the RP23 library (pBACe3.6 vector [45]) were obtained from the BACPAC Resource Center (Children's Hospital Oakland Research Institute, Oakland, California) and maintained in their original host strain DH10B in the presence 12 µg/ml chloramphenicol (unmodified BACs) or 25 µg/ml kanamycin (retrofitted BACs). SelectaBAC retrofitting strategy, adapted from published protocols [27], [46], [47], is described in the Text S1.

### BAC complementation

ESCs maintained on a feeder layer in 12-well plates were transfected with 2 ug of circular BAC DNA using Lipofectamine 2000 Reagent (Invitrogen), according to manufacturer's protocol. Selection was started 48 h later, with the following concentration of drugs maintained for at least 5 days: 1.5 ug/ml puromycin (Sigma), or 150 ug/ml hygromycin (Roche), or 15 ug/ml blasticidin (Sigma), or 30 ug/ml zeocin (Invitrogen). ESC differentiation in embryoid bodies was performed in a LIF-deprived semi-solid media, as described [11]. Genomic DNA from BAC transfected clones was isolated using DNAzol, according to the manufacturer's instructions (Invitrogen). Southern blot detection of transfected BAC DNA was performed using *Eco*RV digestion and a probe specific to the neomycin gene, as described [48]. Total cellular RNA was isolated from BAC transfected clones (undifferentiated ESCs or embryoid bodies) with Trizol (Invitrogen), according to the manufacturer's instructions. Quantitative RT-PCR assays were performed according to standard protocols described in Text S1.

### Generation of chimeric mice

Mouse chimeras were generated by the transgenic facility of IRIC. ESCs [40] corresponding to primary clone no. 9, tertiary clones 9–18 and 9–37 (with *in vitro* phenotype) and BAC-transfected 9–18 and 9–37 clones (rescued *in vitro* phenotype), were injected into C57BL/6 blastocysts or aggregated with CD1 morulas. Tertiary clone 9–18 results showed in Figure 6 are already published [23]. ESCs contribution to chimeric embryos (at E9.5 and E14.5) was evaluated by PCR using genomic DNA extracted with a standard protocol (lysis with of 100 mM NaCl, 10 mM Tris pH 8.0, 25 mM EDTA pH 8.0, 0.5% SDS, and 2.5 ug/ml Proteinase K followed by phenol-chloroform extraction and ethanol precipitation) or Sigma REDExtract-N-Amp Tissue PCR kit (primers specific to the neomycin gene are described in the Table S7). Southern blot analysis was performed as previously described [48] with a neomycin probe and *Eco*RV-digested genomic DNA isolated from E14.5 fetal livers using DNAzol (Invitrogen). ESC contribution to adult mice was determined by evaluation of the coat color chimerism.

### FISH analysis

BACs RP23-143E19 and RP23-323M5 were labeled with Spectrum Orange and Green fluorochromes, respectively, via Nick Translation (Abbott Molecular Cat. No. 32-801300). BACs have been tested both separately and together on mouse control cells from Leukemia Cell Bank of Quebec. A minimum of one hundred interphase nuclei and ten metaphases were evaluated per sample and results are given as signal distribution percentages.

## Supporting Information

Figure S1 Graphical representation of Ki67 values for each puro^S^ tertiary clones grouped in normalized plate sets based on proliferation rate. Puro^S^ tertiary ESC clones presenting similar proliferation rate were arrayed together in 96-well plates: five normalized plate sets were generated (A, B, B*, C, and D) based on the timing of harvest (A = earliest collection, D, latest). Ki67 expressing cells were quantified by flow cytometry. Most tertiary clones presenting <60% Ki67^+^ cells had a slow proliferation rate (e.g. arrayed in plate set D) compared to other clones.
(0.34 MB TIF)

Figure S2 DelES interface. Overview of major functionalities of DelES web database, available online at: http://bioinfo.iric.ca/deles. The *Families* tab is depicted here as an example. It is divided into 2 sub-sections: *Karyoview* and *Families*. The *Karyoview* sub-section provides a graphical representation of the mapped primary virus insertion sites as well as the orientation of the deletions for a given family (color-coded icons, as indicated). Deleted chromosome portions are displayed as red lines to the left of the ideograms. Especially noteworthy is the graphical representation of the genomic context of the deletions. Currently available and customizable tracks are: MGI genes, RP23 library BACs, miRBASE miRNAs, lincRNAs and ultraconserved elements. The *Families* sub-section presents most of the accumulated genetic and phenotypic observations related to puro^S^ tertiary clones. Raw screen data are also accessible from this tab. For more DelES functionalities, see Text S1.
(2.58 MB TIF)

Figure S3 BAC engineering for DelES complementation. (A) The SelactaBAC retrofitting strategy was optimized to introduce a targeting vector (TV) containing a eukaryote (puromycin is depicted) and a prokaryote (kanamycin; Kan) resistance gene into the chroramphenicol (CM) gene of the BAC vector. This protocol relies on the inducible expression (addition of L-arabinose and temperature shift) of λ phage proteins which mediate homologous recombination events between the homology arms of the targeting cassette (identified as A and B) and the BAC vector. Bacteria containing the retrofitted BAC are resistant to kanamycin (Kan^R^) and sensitive to chloramphenicol (CM^S^). (B) Southern blot performed with BAC DNA extracted from bacteria. *Eco*RI restriction digest combined with an external probe hybridizing to the SacB gene of the BAC vector was used to detect proper BAC modification. Fragments of 8.9 kb and 9.8 kb were observed for the unmodified (wt CM^R^) and the modified BACs (Mod. Kan^R^ CM^S^), respectively. Both fragments were observed with BAC DNA extracted from mixed bacterial colonies (contain both modified and unmodified BAC) (C) Southern blot performed with genomic DNA extracted from ESCs stably transfected with a modified BAC. *Nhe*I restriction digest (*Nhe*I sites in both BAC insert and vector) combined with a neo probe revealed a 5.1 Kb and a 21 Kb fragment, corresponding to the integration site of the primary anchor virus and the modified BAC, respectively. (D) Combined relative expression of *Lmnb1*, *Iigp1*, *Isoc1* and *Slc12a2*, in tertiary clone 9–37 following BAC transfections. Values are relative to family 9 primary clone expression levels with standard error (±SE), representative of 2 independent experiments performed in duplicate reactions.
(1.34 MB TIF)

Table S1 Global statistics of DelES library generation.
(1.02 MB TIF)

Table S2 Molecular characterization details of DelES clones used in functional assays. Listing of all DelES clones included in the functional screen grouped in families according to anchor site, and by subfamilies according to Southern blot fragment size. Also included are batch numbers corresponding to normalized plates used for functional screens and a summary of hygromycin detection results evaluated by Southern blot and/or Q-PCR.
(0.18 MB XLS)

Table S3 Summary of deletions distribution. Number of independent deletions, with average deletion size and median per chromosome.
(1.14 MB TIF)

Table S4 Known or predicted haploinsufficient/imprinted candidate genes present in the vicinity of DelES anchor loci that are not associated with chromosomal deletions. Of the 29 families characterized by the absence of puro^S^ or hygro^−^ tertiary clones, 9 that had a minimum of 15 G418^R^ tertiary clones (80^th^ percentile of distribution) were selected for analysis. Genes within a 1.61 Mb window (DelES median deletion size) of each directional anchor site are listed (hypothetical deletions). A literature search was performed to identify candidate genes known or predicted to be haploinsufficient or imprinted (in red). The search for haploinsufficient genes was performed by retrieving all abstracts from Pubmed database that contain “haploinsuff* AND mouse” as a query (August 11^th^, 2009). Then, using a script as reported in [51], a list of haploinsufficient genes was extracted from these selected abstracts. The list of predicted imprinted genes was taken from [52].
(1.20 MB TIF)

Table S5 Genomic content of DelES mapped deletions. List of current DelES mapped families and elements included in the family largest deletion. Clusters (genes, miRNA, ultraconserved elements, lincRNA) are in represented in red and have been identified by manually looking at all mapped families. Number of genes with associated mouse phenotypes has been extracted from MGI [53]. OMIM entries are based on human syntenic regions [54].
(0.10 MB XLS)

Table S6 Embryoid body screen raw data. Table presenting raw data obtained for primary and tertiary clones EB formation screening. EB number were counted manually and seeding densities were obtained by an automated evaluation of the methylene blue stained area.
(0.18 MB XLS)

Table S7 List of oligonucleotides used in all reported assays.
(1.62 MB TIF)

Text S1 Supplemental Materials and Methods.
(0.09 MB DOC)
