# Supplementary figures and images for: Genome-Wide Interrogation of Mammalian Stem Cell Fate Determinants by Nested Chromosome Deletions
Source: PLoS Genet. 2010 Dec 9;6(12):e1001241. doi: 10.1371/journal.pgen.1001241 (PMC3000362; doi:10.1371/journal.pgen.1001241)

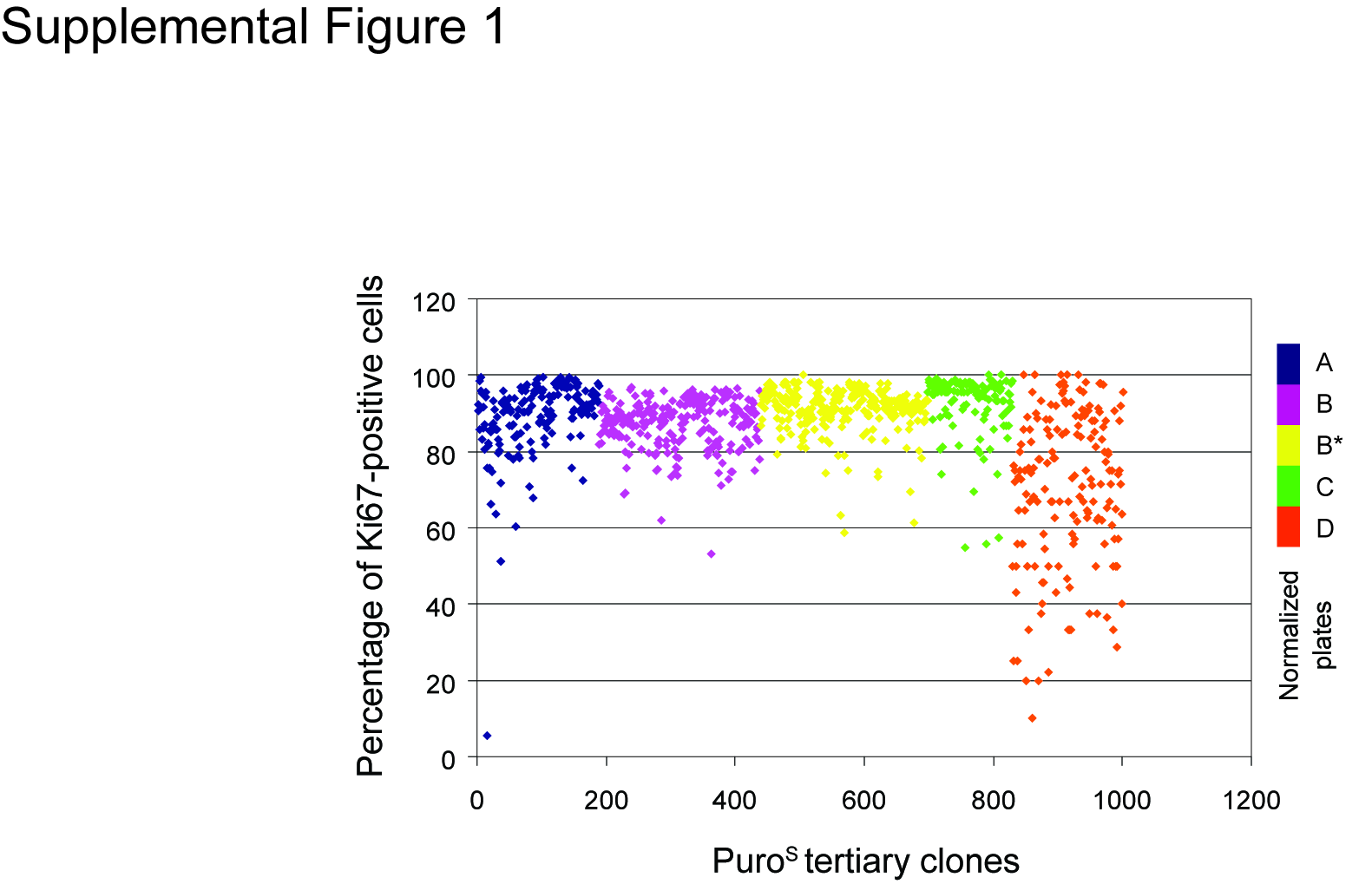

Supplement: Figure S1 — Graphical representation of Ki67 values for each puroS tertiary clones grouped in normalized plate sets based on proliferation rate. PuroS tertiary ESC clones presenting similar proliferation rate were arrayed together in 96-well plates: five normalized plate sets were generated (A, B, B*, C, and D) based on the timing of harvest (A = earliest collection, D, latest). Ki67 expressing cells were quantified by flow cytometry. Most tertiary clones presenting <60% Ki67+ cells had a slow proliferation rate (e.g. arrayed in plate set D) compared to other clones. (0.34 MB TIF) [file pgen.1001241.s001.tif]

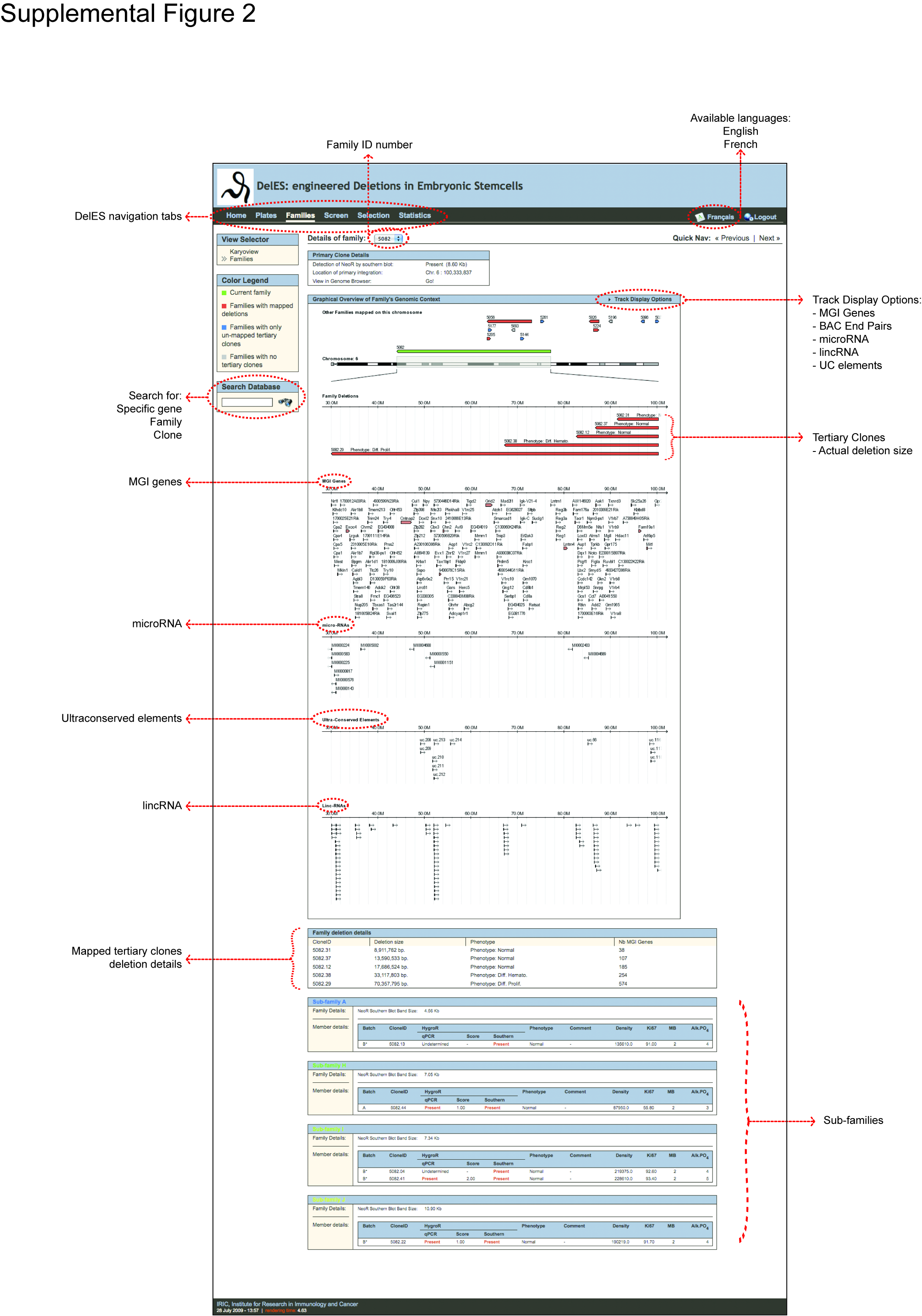

Supplement: Figure S2 — DelES interface. Overview of major functionalities of DelES web database, available online at: http://bioinfo.iric.ca/deles. The Families tab is depicted here as an example. It is divided into 2 sub-sections: Karyoview and Families. The Karyoview sub-section provides a graphical representation of the mapped primary virus insertion sites as well as the orientation of the deletions for a given family (color-coded icons, as indicated). Deleted chromosome portions are displayed as red lines to the left of the ideograms. Especially noteworthy is the graphical representation of the genomic context of the deletions. Currently available and customizable tracks are: MGI genes, RP23 library BACs, miRBASE miRNAs, lincRNAs and ultraconserved elements. The Families sub-section presents most of the accumulated genetic and phenotypic observations related to puroS tertiary clones. Raw screen data are also accessible from this tab. For more DelES functionalities, see Text S1. (2.58 MB TIF) [file pgen.1001241.s002.tif]

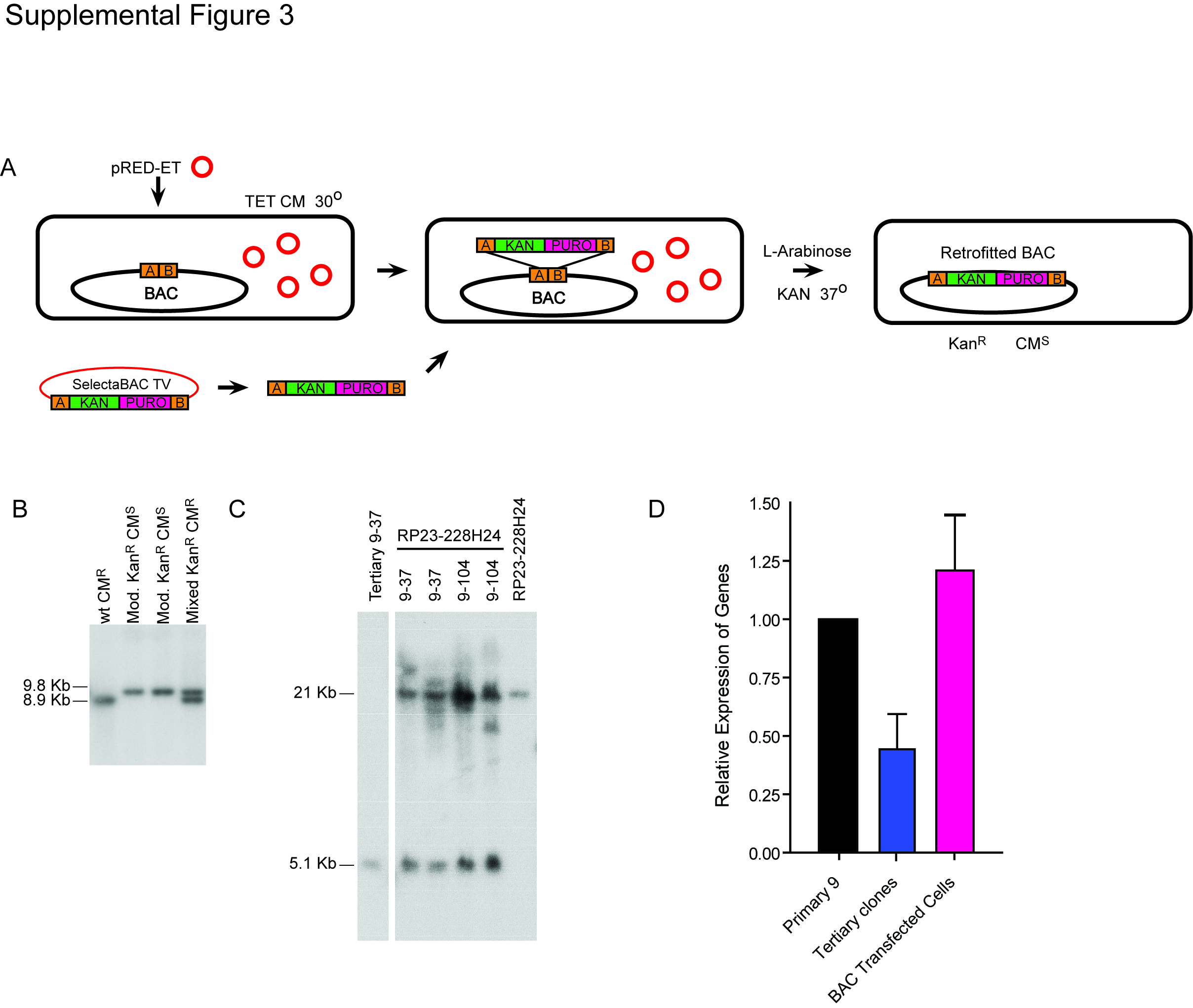

Supplement: Figure S3 — BAC engineering for DelES complementation. (A) The SelactaBAC retrofitting strategy was optimized to introduce a targeting vector (TV) containing a eukaryote (puromycin is depicted) and a prokaryote (kanamycin; Kan) resistance gene into the chroramphenicol (CM) gene of the BAC vector. This protocol relies on the inducible expression (addition of L-arabinose and temperature shift) of λ phage proteins which mediate homologous recombination events between the homology arms of the targeting cassette (identified as A and B) and the BAC vector. Bacteria containing the retrofitted BAC are resistant to kanamycin (KanR) and sensitive to chloramphenicol (CMS). (B) Southern blot performed with BAC DNA extracted from bacteria. EcoRI restriction digest combined with an external probe hybridizing to the SacB gene of the BAC vector was used to detect proper BAC modification. Fragments of 8.9 kb and 9.8 kb were observed for the unmodified (wt CMR) and the modified BACs (Mod. KanR CMS), respectively. Both fragments were observed with BAC DNA extracted from mixed bacterial colonies (contain both modified and unmodified BAC) (C) Southern blot performed with genomic DNA extracted from ESCs stably transfected with a modified BAC. NheI restriction digest (NheI sites in both BAC insert and vector) combined with a neo probe revealed a 5.1 Kb and a 21 Kb fragment, corresponding to the integration site of the primary anchor virus and the modified BAC, respectively. (D) Combined relative expression of Lmnb1, Iigp1, Isoc1 and Slc12a2, in tertiary clone 9–37 following BAC transfections. Values are relative to family 9 primary clone expression levels with standard error (±SE), representative of 2 independent experiments performed in duplicate reactions. (1.34 MB TIF) [file pgen.1001241.s003.tif]

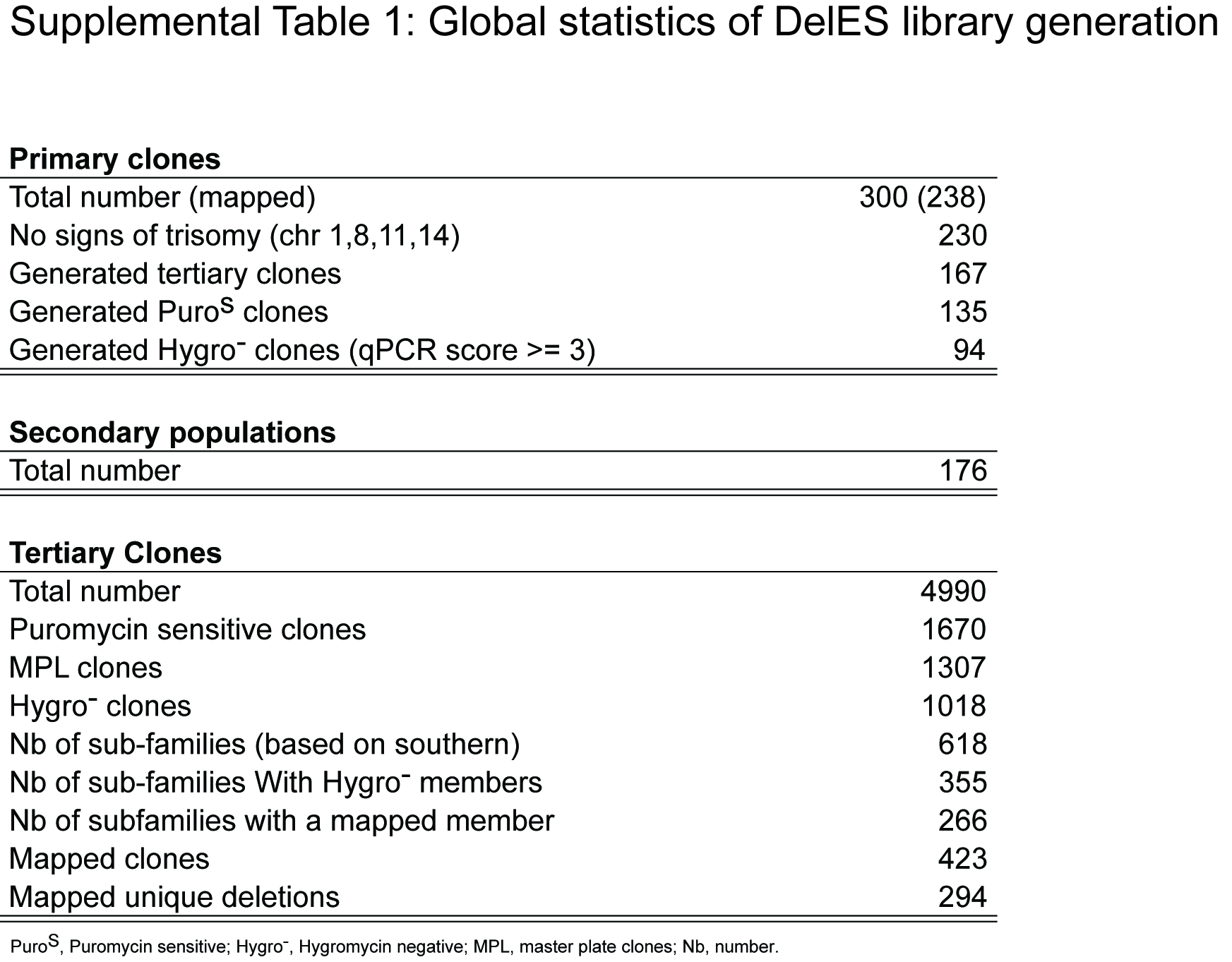

Supplement: Table S1 — Global statistics of DelES library generation. (1.02 MB TIF) [file pgen.1001241.s004.tif]

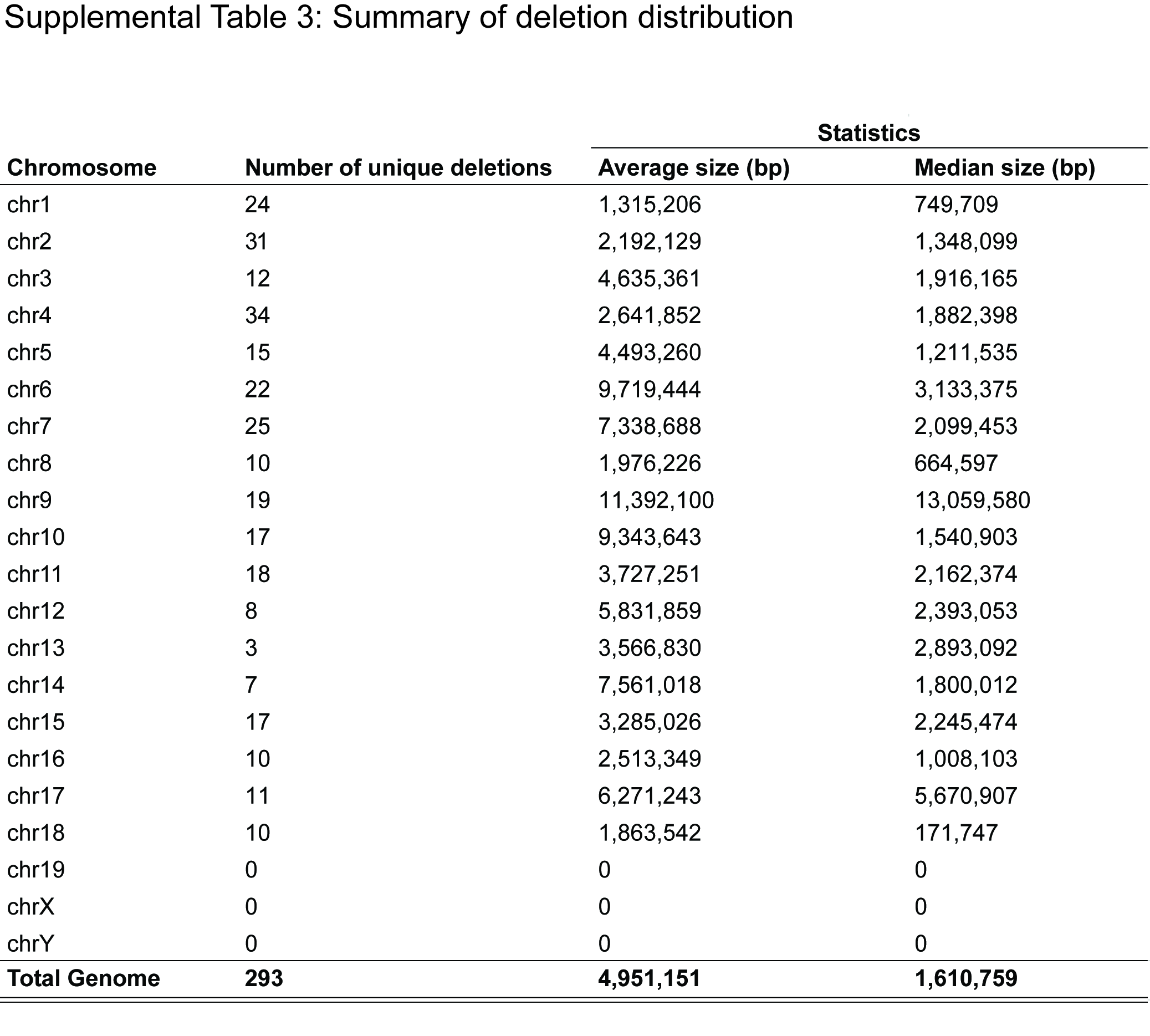

Supplement: Table S3 — Summary of deletions distribution. Number of independent deletions, with average deletion size and median per chromosome. (1.14 MB TIF) [file pgen.1001241.s006.tif]

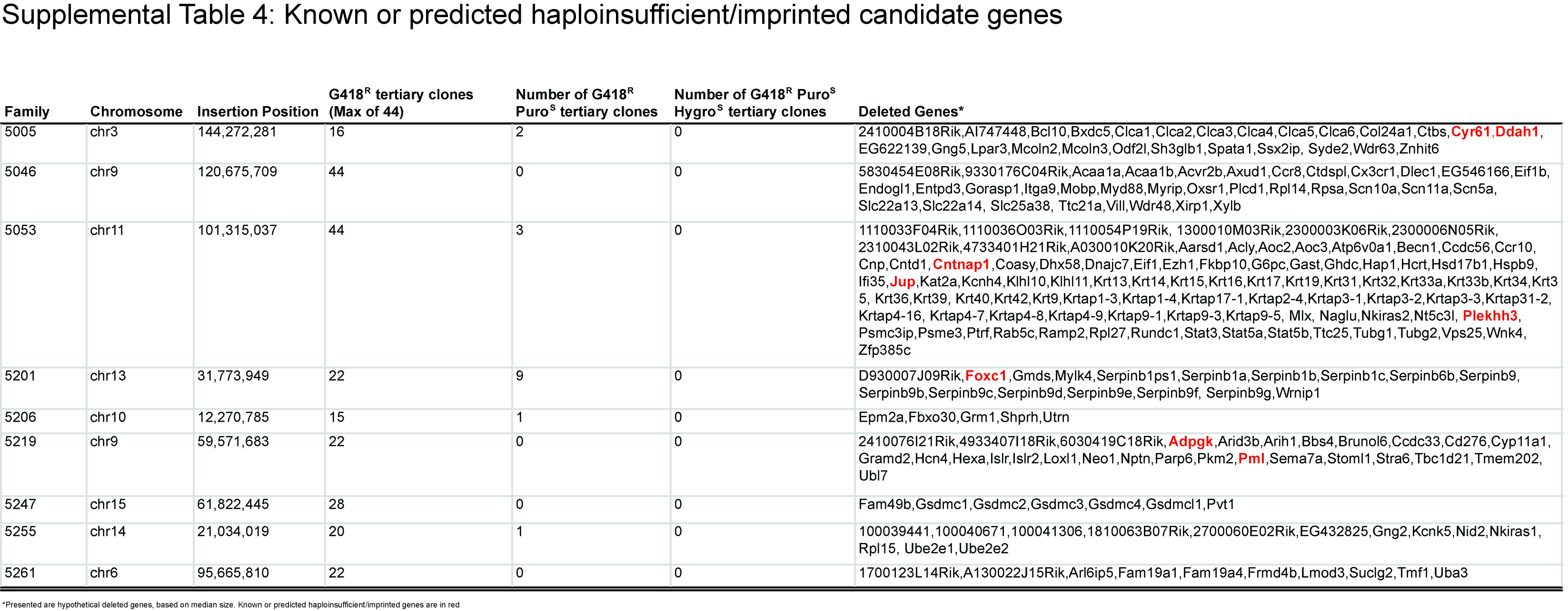

Supplement: Table S4 — Known or predicted haploinsufficient/imprinted candidate genes present in the vicinity of DelES anchor loci that are not associated with chromosomal deletions. Of the 29 families characterized by the absence of puroS or hygro− tertiary clones, 9 that had a minimum of 15 G418R tertiary clones (80th percentile of distribution) were selected for analysis. Genes within a 1.61 Mb window (DelES median deletion size) of each directional anchor site are listed (hypothetical deletions). A literature search was performed to identify candidate genes known or predicted to be haploinsufficient or imprinted (in red). The search for haploinsufficient genes was performed by retrieving all abstracts from Pubmed database that contain “haploinsuff* AND mouse” as a query (August 11th, 2009). Then, using a script as reported in [51], a list of haploinsufficient genes was extracted from these selected abstracts. The list of predicted imprinted genes was taken from [52]. (1.20 MB TIF) [file pgen.1001241.s007.tif]

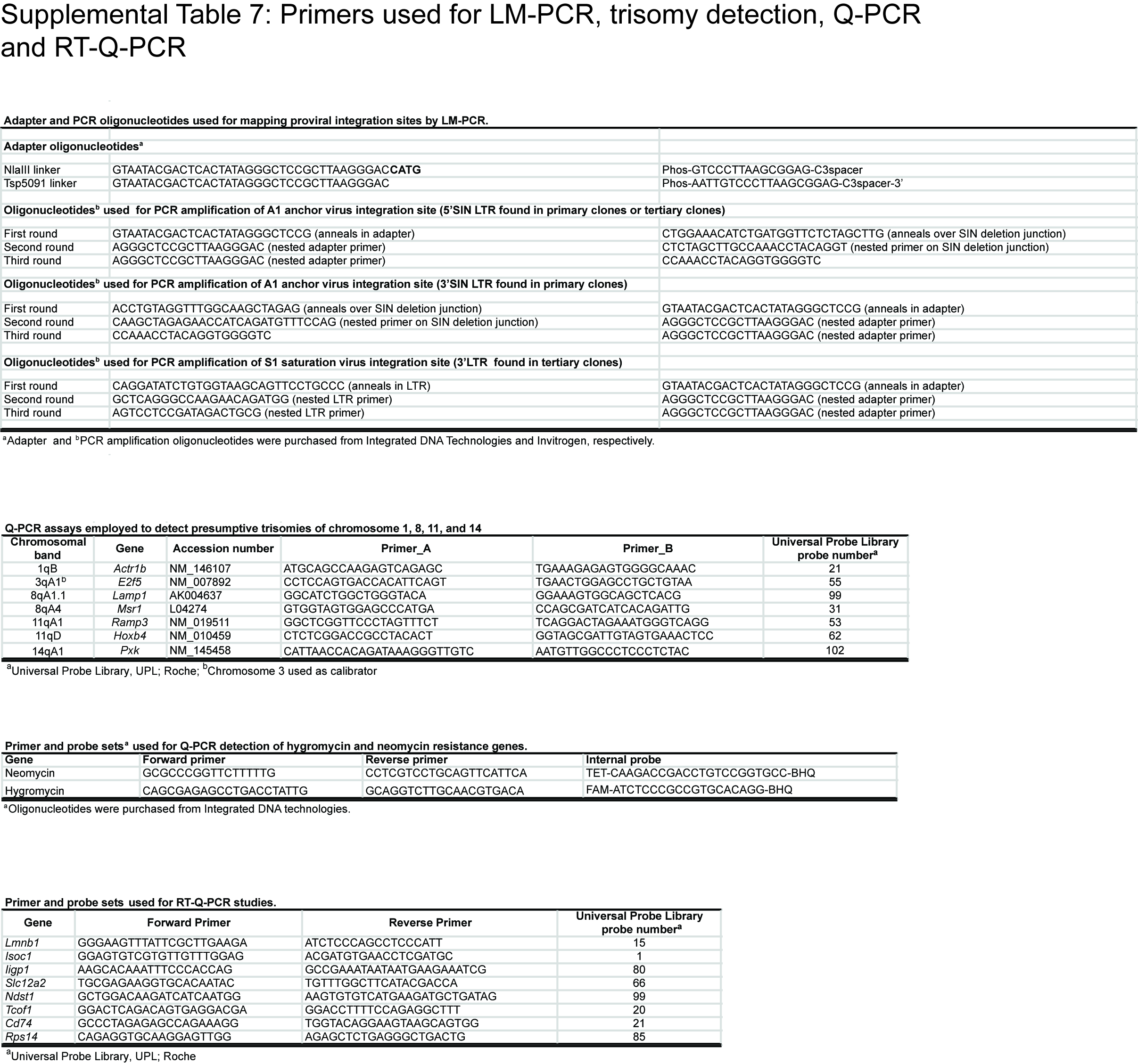

Supplement: Table S7 — List of oligonucleotides used in all reported assays. (1.62 MB TIF) [file pgen.1001241.s010.tif]
