# Supplementary material for: Genome-Wide Interrogation of Mammalian Stem Cell Fate Determinants by Nested Chromosome Deletions
Source: PLoS Genet. 2010 Dec 9;6(12):e1001241. doi: 10.1371/journal.pgen.1001241 (PMC3000362; doi:10.1371/journal.pgen.1001241)
Supplement: Text S1 — Supplemental Materials and Methods. (0.09 MB DOC) [file pgen.1001241.s011.doc]

# SUPPLEMENTAL EXPERIMENTAL PROCEDURES

## DelES library generation

R1 ESCs [1] were infected with low titer of virus A1 (virus-containing supernatant diluted 1:500) and 288 primary ESC clones were isolated following 6-9 days of puromycin selection (1.5 ug/ml, Sigma) (Figure 1C and Supplemental Table 1). Clonal analysis performed by Southern Blot using genomic DNA extracted from primary clones revealed multiple insertions of the A1 virus at low frequency (10 out of 235 analyzed genomic DNA samples). Prior to the second infection (retrovirus S1), primary ESC clones were screened for potential trisomies, which were frequently observed in these culture conditions (e.g. chromosome 1, 8, 11, and 14) (see real-time quantitative PCR section below). From these analyses, 20.1% of primary clones (58 out of 288 analyzed) were rejected (Figure 1B and Supplemental Table 1). Among primary clones that appear free of presumptive trisomy, 176 were independently infected with the S1 saturation virus (virus-containing supernatant diluted 1:12), and then selected with hygromycin (150 ug/ml, Roche) for 7 days, generating the secondary populations used for Cre-electroporation (Figure 1C and Supplemental Table 1). Following 7-9 days of G418 selection (300 ug/ml, Invitrogen), 4929 tertiary clones related to 156 anchor sites were isolated (Figure 1C and Supplemental Table 1). No G418^R^ tertiary clones could be derived from 20 secondary clone populations, mainly for technical reasons. Puromycin sib-selection was conducted for each tertiary clone. An average of 11± 10 (range: 0 to 42 clones) puromycin-sensitive (puro^S^) tertiary clones were collected per family in 96-well plates (n=1670 Cherry-picked clones; CPC clones) (Figure 1C, Supplemental Table 1**)**. From the 156 families of G418^R^ tertiary clones, 21 did not contain puro^S^ tertiary clones (puro^S^ tertiary clones are related to 135 anchor site) (Figure 1C).

A large proportion of puro^S^ tertiary ESC clones (n=1307), containing chromosomal rearrangements anchored to 104 independent loci, were used to conduct preliminary functional screens (Figure 1C and Supplemental Table 1). Puro^S^ tertiary ESC clones presenting similar proliferation rate were arrayed together in 96-well plates, in order to get homogenous cell densities for functional studies. Five normalized plate sets were generated (A, B, B*, C, and D) based on the timing of harvest (A=earliest collection, D, latest) (see Supplemental Table 2 for normalized plate set attribution for each tertiary clone). A robotic cell culture system assisted the expansion of cells in normalized plates and the seeding of the following functional assays: alkaline phosphatase staining, flow cytometry analysis, and colony staining (see *functional assays* sections below). The last column of each plate was left empty in order to add control samples (2 wells each): R1 wild-type ESCs, and tertiary clones 9-35, 9-18, and 9-104. Cells from normalized plates were frozen in 96-wells plates (labeled MPL0xxx) and genomic DNA extracted with DNAzol, according to manufacturer’s instructions (Invitrogen).

## Cryopreservation of cells

At each stage of the pipeline, cells were cryopreserved in liquid nitrogen for archiving of the library (long-term viability confirmed after 3 years of storage). Primary clones and secondary clone populations were individually frozen in 1ml-format CryoTubes^TM^ (NUNC). Puro^S^ tertiary (CPC) clones, maintained in 96-well plates, were individually frozen in cryotubes labeled with a 2D bar code (CryoBank^TM^, NUNC). All tertiary clones (plates labeled TER0xxx) and isolated puro^S^ tertiary clones (plates labeled CPC0xxx) were frozen in 96-well polypropylene plates (Costar, Fisher Scientific) sealed with rubber mats (Fisher Scientific).

## Functional assays

### Alkaline phosphatase detection

Prior to alkaline phosphatase detection, ESCs were maintained on a feeder layers in presence of LIF, as previously described [2]. After dissociating the cells from the normalized plates, the following proportions of cells were used to seed the phosphatase alkaline assay plates: 2%, 4%, and 8% (2%, 6%, and 18% for normalized plate set A only). Following three days of culture, alkaline phosphatase detection was performed with two of these plates, according to the manufacturer’s instructions (Alkaline Phosphatase Detection Kit, Chemicon). A score from 1 (differentiated) to 5 (undifferentiated) was manually assigned to each clone using an inverted microscope.

### Flow cytometry analyses

Cells from tertiary clones, maintained on 96-well gelatinized plates in presence of LIF, were dissociated. Fifty percent of the cells from each well were counted using TruCOUNT reference beads (BD Biosciences) or stained with a PE-conjugated mouse anti-human Ki67 monoclonal antibody (BD Biosciences) and analyzed by flow cytometry. Debris and dead cells were excluded from the analysis using forward and orthogonal light scatter criterias. All the samples were processed using an analytical flow cytometer (BD LSR II, BD Biosciences, San Jose, CA, USA) and data analyzed with FlowJo software (Tree Star Inc., Asland, OR, USA).

### Colony staining

For each tertiary clone present on a 96-well normalized plate, ~15% of the cells dissociated in each well were used to seed a gelatinized 96-well plate. Cells were maintained in presence of LIF for one day. Media was removed and staining was directly performed in the plates with 100ul of methylene blue solution per well (0.3% methylene blue in methanol, Sigma). After 10 minutes incubation at room temperature, plates were washed in water and dried. Scoring was done manually using an inverted microscope (score: 0-no colonie to 5-high cell density) or by automated microscopy (colony number, average size of colonies, and surface of the well covered with cells) using Northern Eclipse software (EMPIX Imaging Inc.).

## PCR studies

**Real-time quantitative PCR (Q-PCR) screening of presumptive trisomies**

Gene copy number was determined using primer and probe sets from Universal ProbeLibrary (Exiqon TaqMan probes, Roche Diagnostics) described in Supplemental Table 6. Gene copy number was evaluated for one gene per chromosome (e.g. chromosome 1, 8, 11, and 14) and normalized to a gene present on chromosome 3, since this chromosome is not frequently involved in mouse ESC trisomy [2,3]. PCR reactions for 384-well plate formats were performed using 2 µl of DNA sample (50 ng), 5 µl of the TaqMan PCR Master Mix (Applied Biosystems, CA), 2 µM of each primer and 1 µM of the Universal TaqMan probe in a total volume of 10 µl. The ABI PRISM® 7900HT Sequence Detection System (Applied Biosystems) was used to detect the amplification level and was programmed to an initial step of 10 minutes at 95˚C, followed by 40 cycles of 15 seconds at 95˚C and 1 minute at 60˚C. All reactions were run in triplicate and the average values were used for quantification. A standard curve was generated for each assay (absolute quantification). Normalization was done according to chromosome 3 assay. A ratio of 1.5 (test versus chromosome 3 control) was indicative of a potential trisomy.

**Q-PCR detection of hygromycin resistance gene**

For detection of the hygromycin resistance gene in gDNA of puro^S^ tertiary clones, duplex Q-PCR reactions were performed with differentially labeled TaqMan probes specific to the hygromycin and neomycin (neo, reference) resistance genes. Threshold cycle (Ct) values for hygro (Ct_hygro_) and neo (Ct_neo_) were compared for each clone (∆Ct= Ct_hygro_-Ct_neo_) and represented by a score from 1 to 5: score 1, ∆Ct ≤ -2; score 2, ∆Ct = -2 to 2; score 3, ∆Ct = 2 to 5; score 4, ∆Ct = 5 to 10; and score 5, ∆Ct > 10. Puro^S^ tertiary clones associated to scores 1-3 or 4-5 were considered positive or negative for hygromycin gene, respectively.

### Mapping of proviral integrations

Integration sites of the anchor virus were mapped in primary clones by I-PCR or LM-PCR. Saturation virus integration sites were mapped in tertiary clones by LM-PCR. The I-PCR approach was previously described [2]. The LM-PCR strategy, which relies on specific oligonucleotides described in Supplemental Table 6, was adapted from a published protocol summarized here [4]. Briefly, 1 ug of gDNA was linearized with *Nla*III or *Tsp*5091 (New England Biolabs) and these restriction enzymes were subsequently heat inactivated. Splinkerette adapters were made by annealing 50μM of complementary oligonucleotides together (5 minutes thermal denaturation followed by gradual cooling). Seventy-five picomoles of splinkerette adapter were ligated to 250ng linearized DNA with 20U T4 DNA ligase (Invitrogen) in a final volume of 20μl at 16ºC overnight followed by heat inactivation. Five microliters of ligation product was used as a template in the first round of PCR, while the second and third rounds of PCR amplified 2ul of previous PCR product diluted 1/50. PCR reactions contained 200μM each dNTPs, 400μM each primer, 2mM MgCl_2_, 1X reaction buffer, and 2,5U of Taq DNA polymerase (Invitrogen), in a total volume of 50μl. PCR was performed using a GeneAmp 9700 thermocyler (Perkin Elmer) with the following program: 94ºC for 5min; 94ºC for 30sec, 56ºC for 30sec, 72ºC for 60sec (25 cycles); 72ºC for 5min; 4ºC hold. Third round PCR DNA products were excised from 2% agarose gel, purified with Qiaquick gel extraction kit (Qiagen), and directly sequenced with AGTCCTCCGATAGACTGCG (3’LTR) or CCAAACCTACAGGTGGGGTC (5’LTR) primer. Vector sequence contaminations were removed with Cross_match (Laboratory of Phil Green, University of Washington, http://www.phrap.org/) and sequences mapped using the BLAT alignment tool of the UCSC Genome Browser ( <http://genome.ucsc.edu/>, NCBI mouse Build 37) [5].

### Q-RT-PCR

Two micrograms of ESC or EB RNA was reverse transcribed using Superscript II Reverse Transcriptase and random hexamers, according to the manufacturer’s guidelines (Invitrogen). Primer and probe sets for target genes (Exiqon probes, Roche Diagnostics) were designed using the Roche Universal ProbeLibrary assay design software (Advanced primer3 settings) (Supplemental Table 6). Gene expression assays were tested for maximum efficiency by standard curve analysis (slope = 3.1 - 3.6) (Applied Biosystems). Reference gene assays (*Gapdh* and *ß-actin*) were purchased from ABI (20X primer-probe mix, VIC labeled). Triplicate reactions were prepared in 384-well plates (5-25 ng of cDNA per reaction) and monitored with the ABI PRISM® 7900HT Sequence Detection System (Applied Biosystems) (see *Real-time quantitative PCR (Q-PCR) screening of presumptive trisomies* section for PCR mix and amplification parameters**)**. Relative quantification of the target genes was calculated with respect to the average level of two calibrator genes, *Gapdh* and *ß-actin*, and normalized against the corresponding mock-transfected primary clone, using the delta-delta Ct method.

## DelES database functionalities

The *Home* tab provides information on the database, supporting grants and Figures detailing the experimental procedures. The *Plates* tab (restricted access) provides information on all the biological material (Cells, DNA, RNA) and their storage location. The *Families* tab has been described in Supplemental Figure 2. The *Screen* tab presents a chromosome view of scored phenotypes and complementation information when available. The *Selection* tab is the main data-mining tool of the web interface and allows users to select DelES families based on a series of predefined parameters such as the presence of clones presenting specific phenotypes, deletions spanning microRNAs, and others. Finally, the *Statistics* tab provides an overview of the percentage of the genome and individual chromosomes covered by the mapped deletions. Some specific information on the library such as the number of primary clones or the number of unique mapped deletions is also present. The most common points of entry of the database are the Karoyview, the Selection tool and the Search box. The Search box, present on most pages of the site, allows users to search the contents of DelES by inputting keywords. Users can initiate a search using clone or plate names or input a gene symbol and find any deletions covering said gene. Since DelES uses a local installation of Ensembl to manage the annotation tracks of the *Families* tab’s genome browser version, users can search DelES using any gene symbol or synonym known to Ensembl Genome Browser [6] (http://www.ensembl.org/index.html).

## SelectaBAC modification strategy

Two homology arms specific to the chloramphenicol resistance gene were generated by PCR and subcloned in pDrive vector (Qiagen), flanking the bacterial kanamycin resistance gene from the vector and an eukaryote resistance marker gene generated by PCR (puromycin, or hygromycin, or blasticidin, or zeocin) regulated by a *Pgk* promoter and a SV40 mRNA polyadenylation signal. The engineered cassettes were then sub-cloned into the pLD53 vector by *Sst*I and *Mlu*I (pDrive)/*Asc*I (pLD53) digestion, thus creating the pSelectaBAC-Puro, -Hygro, -Blast, -Zeo retrofitting vectors, which were maintained in Pir2 E.coli (Invitrogen). The linear targeting cassettes were excised from the plasmid backbone by *Bam*HI + *Sst*I digestion and gel purified for retrofitting. DH10B E.coli containing BACs of interest were made electro-competent by washing fresh mid-log culture twice in ice-cold water and were transformed with the temperature sensitive pRedET plasmid (GeneBridges) and maintained at 30ºC with 3μg/ml tetracycline, thus rendering them recombination proficient. The presence of pRedET in the BAC clones was confirmed by plasmid mini-prep and restriction digests. BAC clones containing pRedET were grown at 30ºC to mid-log, recombination proteins were induced in with 0.33% L-arabinose at 37ºC for 45 minutes. The induced cells were made electro-competent as before and transformed with the linear targeting cassette. Retrofitted BACs were selected on kanamycin (25μg/ml) plates at 37 ºC. Preliminary identification of kanamycin resistant bacterial clones containing modified BACs was performed by submitting each clone to chloramphenicol and ampicillin sib-selection in order to exclude clones containing unmodified BACs (chloramphenicol resistance) and/or persistent targeting vector (ampicillin resistance), respectively. As expected, none of the colonies exhibited resistance to ampicillin, confirming that residual pLD53-based targeting vector was unable to replicate in the BAC host strain. Approximately 65% of kanamycin resistant colonies also exhibited resistance to chloramphenicol (1/10 to 5/10 colonies checked, n=42 experiments). Dual resistant bacterial clones were in fact mixed colonies, possibly due to late recombination events, since single kanamycin or chloramphenicol resistant sub-clones can be isolated from them. Homologous recombination events were confirmed by PCR using primers spanning the homology region and/or by Southern blot using an *Eco*RI enzymatic digestion and an external probe hybridizing to the SacB gene of the BAC vector. Southern blot analysis demonstrated homologous recombination events in all of the kanamycin resistant colonies tested. The absence of non-homologous recombination events was confirmed by comparative DNA fingerprinting of retrofitted and unmodified BACs (*Eco*RI restriction digests). BAC DNA for restriction digests, PCR and Southern blots was prepared with the modified alkaline lysis mini-prep protocol described by the BACPAC Resource Center (http://bacpac.chori.org/). BAC DNA for transfection into ESCs was prepared by modified alkaline lysis followed by purification with NucleoBond AX 500 Tips (Clontech), as described by the University of Michigan Transgenic Animal Model Core [7].

# SUPPLEMENTAL REFERENCES

1. Nagy A, Rossant J, Nagy R, Abramow-Newerly W, Roder JC (1993) Derivation of completely cell culture-derived mice from early-passage embryonic stem cells. ProcNatlAcadSciUSA 90: 8424-8428.

2. Bilodeau M, Girard S, Hebert J, Sauvageau G (2007) A retroviral strategy that efficiently creates chromosomal deletions in mammalian cells. Nat Methods 4: 263-268.

3. Liu X, Wu H, Loring J, Hormuzdi S, Disteche CM, et al. (1997) Trisomy eight in ES cells is a common potential problem in gene targeting and interferes with germ line transmission. Dev Dyn 209: 85-91.

4. Mikkers H, Allen J, Knipscheer P, Romeijn L, Hart A, et al. (2002) High-throughput retroviral tagging to identify components of specific signaling pathways in cancer. Nat Genet 32: 153-159.

5. Kent WJ (2002) BLAT--the BLAST-like alignment tool. Genome Res 12: 656-664.

6. Hubbard TJ, Aken BL, Ayling S, Ballester B, Beal K, et al. (2009) Ensembl 2009. Nucleic Acids Res 37: D690-697.

7. Van Keuren ML, Gavrilina GB, Filipiak WE, Zeidler MG, Saunders TL (2009) Generating transgenic mice from bacterial artificial chromosomes: transgenesis efficiency, integration and expression outcomes. Transgenic Res 18: 769-785.
